# Abscisic Acid Regulates Auxin Distribution to Mediate Maize Lateral Root Development Under Salt Stress

**DOI:** 10.3389/fpls.2019.00716

**Published:** 2019-06-05

**Authors:** Chongchong Lu, Mo-Xian Chen, Rui Liu, Lin Zhang, Xuanxuan Hou, Shouxu Liu, Xinhua Ding, Yong Jiang, Jiandi Xu, Jianhua Zhang, Xiangyu Zhao, Ying-Gao Liu

**Affiliations:** ^1^State Key Laboratory of Crop Biology, Shandong Agricultural University, Tai’an, China; ^2^Department of Biology, Hong Kong Baptist University, Shatin, Hong Kong; ^3^State Key Laboratory of Agrobiotechnology, The Chinese University of Hong Kong, Shatin, Hong Kong; ^4^School of Life Sciences, Shandong University, Jinan, China; ^5^Department of Agronomy, Northeast Agricultural University, Harbin, China; ^6^National Oceanography Centre, Qingdao, China; ^7^Rice Research Institute, Shandong Agricultural Research Institute, Jinan, China

**Keywords:** hormone regulation, root initiation, seedling, salt stress, *Zea mays*

## Abstract

Roots are important plant organs. Lateral root (LR) initiation (LRI) and development play a central role in environmental adaptation. The mechanism of LR development has been well investigated in *Arabidopsis*. When we evaluated the distribution of auxin and abscisic acid (ABA) in maize, we found that the mechanism differed from that in *Arabidopsis*. The distribution of ABA and auxin within the primary roots (PRs) and LRs was independent of each other. Auxin localization was observed below the quiescent center of the root tips, while ABA localized at the top of the quiescent center. Furthermore, NaCl inhibited LRI by increasing ABA accumulation, which mainly regulates auxin distribution, while auxin biosynthesis was inhibited by ABA in *Arabidopsis*. The polar localization of ZmPIN1 in maize was disrupted by NaCl and exogenous ABA. An inhibitor of ABA biosynthesis, fluridone (FLU), and the ABA biosynthesis mutant *vp14* rescued the phenotype under NaCl treatment. Together, all the evidence suggested that NaCl promoted ABA accumulation in LRs and that ABA altered the polar localization of ZmPIN1, disrupted the distribution of auxin and inhibited LRI and development.

## Introduction

Roots constitute the first organ to respond to environmental changes and are essential for sessile organisms in terms of anchoring to the soil and taking up nutrients and water (Aiken and Smucker, 1996; Benkova and Bielach, 2010; Xing et al., 2016). Root formation can be separated into primary, lateral and adventitious roots (Atkinson et al., 2014). Lateral roots (LRs) are devoted to water-use efficiency and the absorption of macronutrients from the surrounding environment, and LR development is tightly regulated by the environment and hormones (Casimiro et al., 2003; Cuesta et al., 2013; Duan et al., 2013). LRs originate from a differentiated layer of cells called founder cells in the pericycle of primary roots (PRs) in *Arabidopsis* and in xylem cells and cells near protophloem vessels in maize (Hochholdinger et al., 2004; Benkova and Bielach, 2010; Lavenus et al., 2013; Orman-Ligeza et al., 2013; Yu et al., 2016). In *Arabidopsis*, there are eight developmental stages from lateral root primordia (LRP) to LRs and three important phases of the LR life cycle: initiation, formation of LRP, and post-emergence growth (Casimiro et al., 2003; Orman-Ligeza et al., 2013).

Previous research has indicated that LR development is controlled by many extrinsic (environment and nutrition) and intrinsic signals (hormones and signaling molecules) in *Arabidopsis* and cereals (Peret et al., 2009; Lavenus et al., 2013; Orman-Ligeza et al., 2013; Atkinson et al., 2014). Salt treatment inhibits PR elongation, LR growth, root hair formation, and root tropism (He et al., 2005; Sun et al., 2008; Wang et al., 2009; Galvan-Ampudia et al., 2013). Plant hormones, including auxin, cytokinins, jasmonic acid, ethylene, and abscisic acid (ABA), play central roles in these processes (De Smet et al., 2006; Peret et al., 2009; Orman-Ligeza et al., 2013; Atkinson et al., 2014). The function of auxin in LR development is especially well investigated (Ditengou et al., 2008; Lavenus et al., 2013). Previous studies have suggested that auxin transport, biosynthesis and signaling regulate lateral root initiation (LRI) and LRP development in *Arabidopsis* (Benkova et al., 2003; Peret et al., 2009; Lavenus et al., 2013). Auxin response mutants have been used to show that some genes, such as *IAA28*, *ARF5*, *ARF7*, and *ARF19*, are involved in priming auxin-regulated LR founder cells and controlling the cell cycle and properties of overlying cells (Goh et al., 2012). Auxin carriers such as *AUX1* and *PIN*s have also been reported to optimize auxin supplies and support LRI. PINs mainly play essential functions in transporting intercellular auxin, and their localization regulates the gravitropism of roots (Xi et al., 2016). ZmPIN1a especially plays a key regulatory role in auxin transport and promotes auxin transport from the stem to the roots (Li et al., 2018). If the distribution of endogenous auxin in LR founder cells is altered by the inhibition of polar auxin transport, LRI is sufficiently blocked (Casimiro et al., 2001; Lavenus et al., 2013; Marhavy et al., 2013). In maize, auxin also plays a pivotal role in LR development. Previous results indicate that *rum1* mutants, whose mutation affects auxin transport, fail at initiating LRs (Gallavotti et al., 2008; Atkinson et al., 2014; Zhang et al., 2014). As a transcriptional activator, LATERAL ROOT PRIMORDIA 1 (LRP1) also participates in this process and has been reported to act downstream of auxin/indole-3-acetic acid (IAA) genes (Zhang et al., 2015). The results of recent studies also indicate that the auxin efflux carrier P-glycoprotein (ZmPGP1) plays an important role in the aluminum (Al)-based regulation of auxin distribution in maize (Zhang et al., 2018). Al stress is associated with reduced auxin accumulation in maize root tips. In contrast, Al stress induces the accumulation of auxin in *Arabidopsis* root tips, a process that is regulated by ZmPGP1, and thus inhibits root growth (Yang Z.B. et al., 2017; Zhang et al., 2018). However, the effects of auxin distribution on LR development and the mechanism by which auxin distribution is regulated under salt stress are still unknown in maize.

ABA is considered a plant stress hormone (Zhu, 2002; Nambara and Marion-Poll, 2005; Nakashima et al., 2006). ABA was recently reported to take part in the regulation of LR development (De Smet et al., 2006; Ding and De Smet, 2013; Duan et al., 2013; Xing et al., 2016). Relatively high concentrations of ABA inhibit both PR and LR growth, while PRs are inhibited less severely than LRs under low concentrations of ABA (Ding et al., 2015; Xing et al., 2016). *Arabidopsis* ABA receptor mutants (*pyl8* and *pyl9*) under ABA treatment have significantly increased numbers of LRs (Xing et al., 2016). In addition, ABA biosynthesis and signaling promote LR quiescence under salt stress (De Smet et al., 2006; Duan et al., 2013). ABA also plays a central role in environment-regulated LR development (Ding and De Smet, 2013; Duan et al., 2013; Ding et al., 2015). Previous studies in *Arabidopsis* have indicated that ABA and salt stress affect LR emergence and growth but not initiation (De Smet et al., 2003; Duan et al., 2013; Julkowska et al., 2014). Although the regulation of LR development by ABA is well investigated in *Arabidopsis*, the mechanism by which ABA regulates LR development in maize has seldom been investigated.

The hormones auxin and ABA are involved in LR regulation, and some crosstalk occurs between them (Atkinson et al., 2014). Previous studies have shown that ABA combined with PYL8 functions as a promoter of post-emergence LR growth via an auxin-dependent pathway (Xing et al., 2016). PYL8 directly interacts with MYB77, which can interact with ARF7 to regulate the expression of auxin-induced genes, such as LBD16 and LBD29, to promote LR formation and elongation in *Arabidopsis* (Okushima et al., 2005; Wilmoth et al., 2005; Atkinson et al., 2014). Compared with PYL8, PYL9 also interacts with MYB77 but via a different pathway to regulate LR; relatively high concentrations of auxin can overcome the *pyl8/pyl9*-induced quiescence in *Arabidopsis* (Xing et al., 2016). By affecting the expression of the auxin efflux carrier protein PIN1, ABI4, an ABA-regulated AP2 domain transcription factor (TF), reportedly regulates auxin transport (Shkolnik-Inbar and Bar-Zvi, 2010). Although some research has suggested that the crosstalk of ABA and auxin plays a pivotal role in the regulation of LR development, the relationship between these two hormones is still unclear. For example, auxin can rescue the inhibitory effects of ABA on LR elongation but not on LRI; The interactions between ABA and auxin occur via different regulatory pathways in PRs and LRs. The relationship between these two hormones and LR regulation still needs to be investigated.

Root architecture plays a crucial role in minimizing the effects of stress on plants, with roots proliferating in soil patches that have the highest concentration of nutrients and water and avoiding dry or saline patches (Galvan-Ampudia et al., 2013; Yang et al., 2014). As such, we were interested in analyzing the mechanism of LR arrest in response to NaCl in maize. With this knowledge, we can identify ways to improve the adaptation of maize plants to high-salt environments. For this purpose, we used two transgenic plants, *pZmPIN1a*::*ZmPIN1a*:*YFP* and *DR5rev*::*mRFPer*, to evaluated the dynamic changes in auxin in response to salt treatment in maize (Gallavotti et al., 2008). Moreover, an excursive staining technique for ABA was used to determine the concentration and distribution of ABA during salt treatment (Schraut et al., 2004; Peng et al., 2006; De Diego et al., 2013; Ondzighi-Assoume et al., 2016). These results were analyzed to determine how ABA and auxin are involved in regulating LR development in high-salt environments and the relationship between them.

Our results showed that both PRs and LRs are inhibited by increased NaCl concentrations in maize. ABA accumulates in response to NaCl treatment, especially in the LRI zone and in LRs, and a relatively high concentration of ABA disrupts the localization of PIN1, which regulates the distribution of auxin. The change in the PIN1 distribution subsequently led to a lack of auxin at the tips of the maize LR, which in turn inhibited LR growth. Thus, we hypothesize that, under salt stress, ABA regulates the distribution of PIN1 and subsequently affects the distribution but not the concentration of auxin. The absence of auxin in the root tips triggers its biosynthesis. Although the auxin level increased in the salt-treated maize roots, it also accumulated in the other parts of the LRs but did not promote root growth.

## Materials and Methods

### Plant Material, Growth Conditions, and Treatment

The *vp14* mutant (W22 background) was obtained from the Iowa Stiff Stalk Synthetic heterotic group (Tan et al., 1997; Tai et al., 2016), and *pZmPIN1a*::*ZmPIN1a*:*YFP* and *DR5rev*::*mRFPer* (B73 background) transgenic lines were donated by the Jackson lab (Gallavotti et al., 2008). First, all seeds used in the experiment were surface sterilized with 5% sodium hypochlorite. The seeds were then placed in a round petri dish (diameter = 10 cm) and covered with sterile, moist absorbent cotton gauze for germination. After 3 days, the B73 seedlings with similar root lengths were transferred to 1/2-strength Hoagland’s nutrient solution (HNS) supplemented with either NaCl, ABA (Sigma), fluridone (FLU; Sigma), or diniconazole (DZ; Sigma), after which the seedlings were grown for an additional 4 days (bio-protocol) (Yang L. et al., 2017). Eight hundred milliliters of HNS was then added to a 1000 mL plastic beaker. The seedlings were placed in 1.5 mL centrifuge tubes whose bottoms were removed, which were then placed on a floating plate. With respect to the FLU, ABA, IAA, and DZ treatments, the seedlings were grown in cylindrical glass barrels that were covered with foil to keep the nutrient solutions in the dark to avoid degradation of the treatment solutions. All of the seedlings were placed under the conditions in which the photoperiod was 16 h/8 h light/dark, the temperature was 25°C, and the relative humidity was 60–70%. The concentration gradient of the salt treatments was as follows: 50, 75, 100, 125, and 200 mM NaCl. Other treatment concentrations were as follows: 5 μM FLU; 50 μM DZ; 0.1 μM, 1 and 10 μM IAA; and 1, 5, 20, and 100 μM ABA.

### PR Length and LR Growth Analysis

The seedlings were transferred to 1/2-strength HNS supplemented with different treatment components (NaCl, ABA, FLU, DZ, IAA) and grown for an additional 4 days. With respect to the NaCl gradient concentration treatments, the seedlings were grown in petri dishes for 3 days, and their initial roots were measured. The plants were then transferred to HNS supplemented with 50, 75, 100, 125, or 200 mM NaCl and grown for 4 days. Root length was measured from the top portion of the proximal hypocotyl to the root tip. The number of LRs was counted daily.

### Immunofluorescence and Confocal Microscopy

Immunofluorescence analysis was conducted according to the method described by Ondzighi-Assoume, with modifications (Ondzighi-Assoume et al., 2016). First, the maize roots were cleaned with deionized water. Second, transverse sections of the LRs 2–5 cm below the hypocotyl were acquired by hand sectioning, after which the transverse sections were placed in a stationary liquid of 10 mM sodium phosphate-buffered saline (PBS, pH = 7.2) that contained 0.2% (v/v) glutaraldehyde (GLU, Sigma), 4% (w/v) paraformaldehyde (PFA, Sigma), and 2% (w/v) 1-ethyl-3-(3-dimethylaminopropyl) carbodiimide hydrochloride (EDC, Sigma). The samples were vacuum infiltrated for 2 h and then shaken at 4°C overnight. The root sections were washed three times with 10 mM PBS (pH 7.2) and then hyalinized in 10 mM PBS, which included 0.2% (w/v) cellulase, 0.2% (w/v) pectinase, 3% (w/v) non-fat dried milk and 0.1% Triton X-100 (pH 7.2), for at least 1 h. The root sections were washed again and incubated with 1/2000 anti-ABA polyclonal antibodies (rabbit anti-ABA antibody, Cat#: ABIN334625^1^) on a shaker at 4°C overnight. The samples were washed until they became clear and were transferred to a one drop/mL incubation buffer of goat anti-rabbit IgG secondary antibody (Invitrogen) conjugated to Alexa Fluor 488 (excitation at 488 nm, emission at 505–530 nm) for at least 2 h at room temperature (RT). The transverse sections of the LRs were washed with 0.01 mM PBS and blocked with Citifluor AF1 (Ted Pella, Inc.). A Zeiss LSM880 (Zeiss) confocal laser-scanning microscope was ultimately used for microscopic imaging. Via an argon laser, green fluorescent protein (GFP) was excited at a wavelength of 488 nm and measured at 505–530 nm with a bandpass filter (green). The yellow fluorescent protein (YFP) excitation wavelength was 488 nm, and the detection wavelength was 505–530 nm with a bandpass filter (green). The red fluorescent protein (RFP) excitation wavelength was 561 nm, and the detection wavelength was 600–650 nm with a bandpass filter (red).

### Liquid Chromatography-Mass Spectrometry (LC-MS) for ABA Determination

Fresh roots (200–500 mg) were harvested after NaCl treatment for 2 days. Afterward, the roots were immersed directly in liquid nitrogen and ground to a powder. The powder was then pooled (100–200 mg) and placed into a 1.5 mL centrifuge tube, and 750 μL of freeze solution A [methanol/water/acetic acid (89/10/1 v/v/v)] containing 30 ng of ^2^H-ABA [(-)-5,8′,8′,8′-d4 ABA] was added (Van Gijsegem et al., 2017). After thorough vortexing, each sample was centrifuged at 13000 rpm for 10 min. The supernatant was placed into a new 1.5 mL centrifuge tube, and 450 μL of solution B [methanol/water/acetic acid (89/10/1 v/v/v)] was added to the precipitate, after which each sample was vortexed thoroughly for 4 h. The samples were then centrifuged at 13000 rpm for 10 min, after which the supernatant was combined with the previous supernatant. The mixed supernatant was used to quantify the ABA contents via an LC-MS system (Ultimate TSQ Quantia, Thermo Fisher Scientific).

### Co-localization of ABA and Auxin

All of the *DR5rev::mRFPer* (B73 background) transgenic line seedlings were grown in 1/2-strength HNS for 3 days, after which they were transferred to HNS supplemented with 200 mM NaCl treatment for 12 h. The transverse root sections were then used to observe the fluorescence of DR5-RFP. The root sections were subsequently fixed into the stationary liquid for immediate ABA immunofluorescence, and the two images were ultimately superimposed with Adobe Photoshop.

### RNA Extraction and Quantitative RT-PCR (RT-qPCR)

RNA was extracted from 2- and 3-day-old roots and was used for RT-qPCR. Whole RNA was extracted using an RNAprep Pure Plant Kit (Tiangen). cDNA for RT-qPCR was reverse transcribed from 1000 ng of whole RNA using a TransScript One-Step gDNA Removal and cDNA Synthesis SuperMix and oligo(dT) primers (TransGen). Real-time qPCR analysis was performed using a SYBR Premix Ex Taq^TM^ II (TaKaRa) on a CFX96 Real-Time PCR detection system (Bio-Rad, Munich, Germany) for each of the four biological replicates and three technical replicates. The gene-specific primers used are listed in Supplementary Table S1.

### Statistical Analysis

For every treatment, at least 10 roots were analyzed; all experiments in this study were repeated at least three times. SigmaPlot 12.5 (64 bit) was used to construct histograms. All the results are provided as the means ± standard errors (SEs), and Student’s *t*-tests (*P* < 0.05) were used for statistical analyses.

### Cluster Analysis

Gene Cluster 3.0 and Java Tree View were used to generate gene expression heat maps, which were based on log2-transformed reads per kilobase per million mapped reads (RPKM)/fragments per kilobase of transcript per million mapped reads (FPKM) values (Tai et al., 2016), for genes related to auxin biosynthesis, transport and response and ABA biosynthesis, degradation and response.

### Quantification of Fluorescence Intensity

Images of the roots were obtained by confocal microscopy (Wang et al., 2012), and ImageJ software^2^ was used to analyze the relative fluorescence intensity of the PRs and the transections of roots and LRs. The experiments were repeated at least five times, and at least ten roots were measured each time.

### Accession Numbers

The cDNA sequence data were obtained from the Maize Genetics and Genomics Database (MaizeGDB^3^) and the GenBank database^4^, and the accession numbers are as follows: ZmTAR1, GRMZM2G127160_P01; ZmVT2, GRMZM2G127308_P01; Zm YUC2, GRMZM2G159393_P01; ZmYUC3, GRMZM2G107761_ P01; ZmYUC1, GRMZM2G091819_P01; ZmYUC4, GRMZM2G 141383_P01; ZmYUC5, GRMZM2G132489_P01; ZmYUC6, GRMZM2G019515_P01; ZmYUC7, GRMZM2G480386_P01; ZmYUC8, GRMZM2G017193_P01; ZmPIN1a, GRMZM2G0 98643_P01; ZmPIN2, GRMZM2G074267_P01; ZmAUXIN1, GRMZM2G129413_P01; ZmPIN10a, GRMZM2G126260_P01; ZmPIN10b, GRMZM2G160496_P01; ZmIAA1, GRMZM2G137 367_P01; ZmARF25, GRMZM2G317900_P01; ZmARF34, GRMZM2G160005_P01; ZmAO1, GRMZM2G141535_P01; ZmVP14, GRMZM2G014392_P01; ZmZEP, GRMZM2G127 139_P01; ZmABI1, GRMZM2G300125_P01; ZmABI2, GRMZ M2G018485_P01; ZmABI3, GRMZM2G133398_P01; ZmABI5, GRMZM2G320754_P01; ZmABH1, GRMZM2G179147_P01; and ZmABH4, GRMZM2G065928_P01.

## Results

### NaCl Treatment Inhibits Root Development in Maize

**FIGURE 1:**
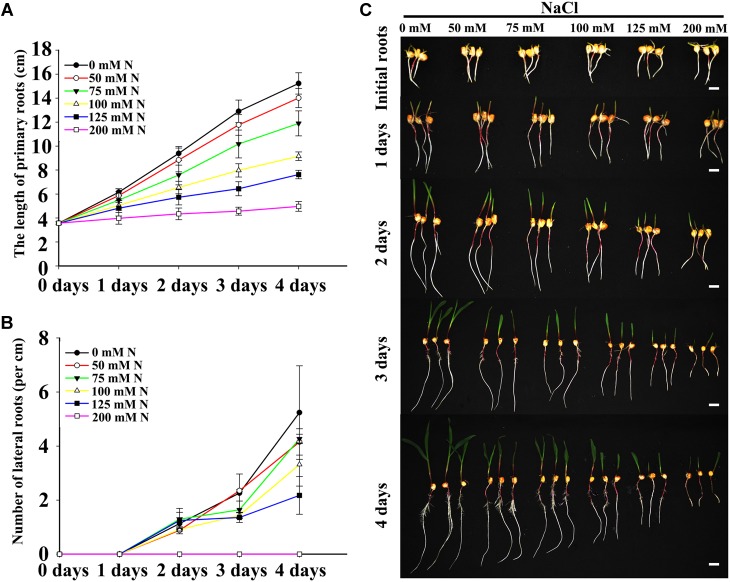
Salt stress inhibited LR and PR development. **(A)** Comparison of the PR length of wild-type (B73) seedlings after imbibition with 0, 50, 75, 100, 125, or 200 mM NaCl. N, NaCl. **(B)** Comparison of the LR density of wild-type (B73) seedlings under 0, 50, 75, 100, 125, or 200 mM NaCl treatment. N, NaCl. **(C)** Phenotypes of wild-type (B73) seedlings after imbibition with 0, 50, 75, 100, 125, or 200 mM NaCl. Bars = 1 cm. The data represent the means ± SEs of five replicates, with 10 seedlings per treatment.

Two fundamental parameters of root system architecture (PR growth rate and LR number and density) were measured or calculated to determine the effects of NaCl on root development. Previous studies have shown that the presence of high concentrations of NaCl in the media inhibit PR growth and LR density in *Arabidopsis* (Duan et al., 2013), and our results also showed that the effects of NaCl on maize roots were associated with the dose (Figure 1). Relatively low concentrations of NaCl (50 and 75 mM) inhibited PR growth and decreased LR density slightly (Figure 1A,B), while the inhibitory effects of NaCl on PR and LRs were more obvious with increasing NaCl concentrations. Under 200 mM NaCl, PR growth was inhibited dramatically, and no visible LRs were detected after an additional 4 days post-transfer (Figure 1A,B and Supplementary Figure S1). Therefore, 100 and 200 mM NaCl were selected for subsequent experiments as appropriate doses to represent moderate and strong salt stresses, respectively.

### NaCl Enhances ABA Accumulations in Maize Roots

**FIGURE 2:**
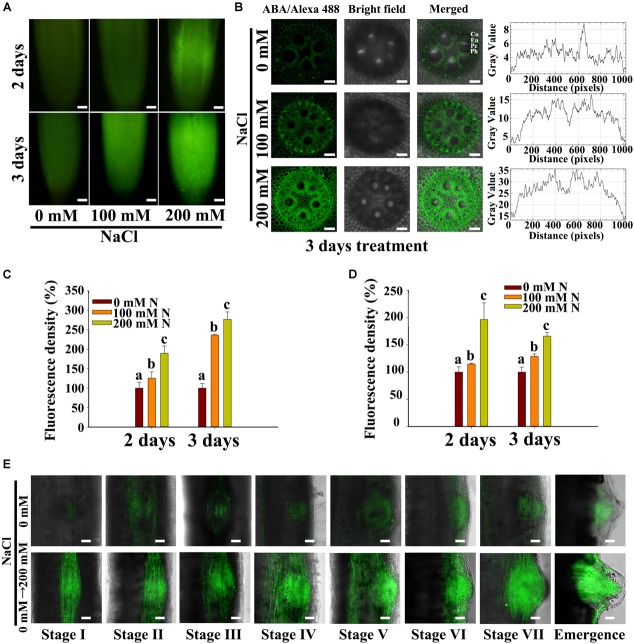
Salt stress stimulated the accumulation of endogenous ABA in the root tips and LRs. **(A)** Observations of the distribution of ABA accumulation by immunofluorescence in PRs subjected to 0, 100, and 200 mM NaCl via confocal microscopy. Bars = 100 μm. **(B)** Observations of ABA localization by immunofluorescence in transverse root sections in the presence of 0, 100, and 200 mM NaCl under confocal microscopy. The curve on the right of the picture shows the ABA/Alexa Fluor 555 fluorescence intensity. En, endodermis; Pc, pericycle; Ph, Phloem. Bars = 50 μm. **(C)** Fluorescence intensity (%) of fluorescence portion in picture **(A)**. The data represent the means ± SEs of five replicates, with 10 seedlings each. N, NaCl. **(D)** Fluorescence intensity (%) of the fluorescence part in picture **(B)**. The data represent the means ± SEs of five replicates, with 10 seedlings each. N, NaCl. **(E)** Observations via confocal microscopy of the ABA distribution and concentration at different developmental stages of LRs subjected to 0 mM or 200 mM NaCl treatment for 8 h. Bars = 50 μm. The figures were selected from five replicates, with 10 seedlings per replicate. The different letters represent significant differences (*P* < 0.05, based on Student’s *t*-test).

Abscisic acid has been reported to promote LR quiescence under salt stress in *Arabidopsis* (Duan et al., 2013). In addition, ABA may also be induced by salt stress in maize roots. Some genes that are reportedly involved in the ABA pathway in maize were chosen for expression analysis. We found that the transcription levels of genes involved in ABA biosynthesis (*ZmAO1*, *ZmVP14*, and *ZmZEP*) (Tan et al., 1997; Lu et al., 2013), ABA catabolism (*ZmABH1* and *ZmABH4*) and ABA signaling (*ZmABI3, 4, 5*) (Fan et al., 2016) significantly increased in response to NaCl treatment (Supplementary Figure S2). We observed a 1/9-fold increase in ABA content in the roots under 100/200 mM NaCl treatment compared with the control treatment (Supplementary Figure S3B). These results implied that ABA might accumulate in roots during the NaCl treatments. Thus, we used the latest immunofluorescence technique to measure changes in ABA content and localization within the roots (Ondzighi-Assoume et al., 2016). We found that, under NaCl treatment, ABA accumulated in the PRs, and more ABA accumulated in response to treatment with 200 mM NaCl than in response to that with 100 mM NaCl (Figure 2A–D). However, the content of ABA increased, and the distribution expanded to all tissues of the roots in response to treatment with NaCl (Figure 2B). We also measured ABA accumulation in LRs and root tips, and the results showed that ABA localized primarily in the tips of the roots and the LRs and that less accumulation occurred in LRP (Figure 2A,E). The transverse root sections showed that ABA is distributed mainly in the endodermis, pericycle and phloem (Supplementary Figure S3). Additionally, ABA accumulated in the LRs during all stages of LR development, and the accumulation was increased by NaCl treatment (Figure 2E).

### ABA Is Involved in the NaCl-Induced Inhibition of LR Emergence in Maize

**FIGURE 3:**
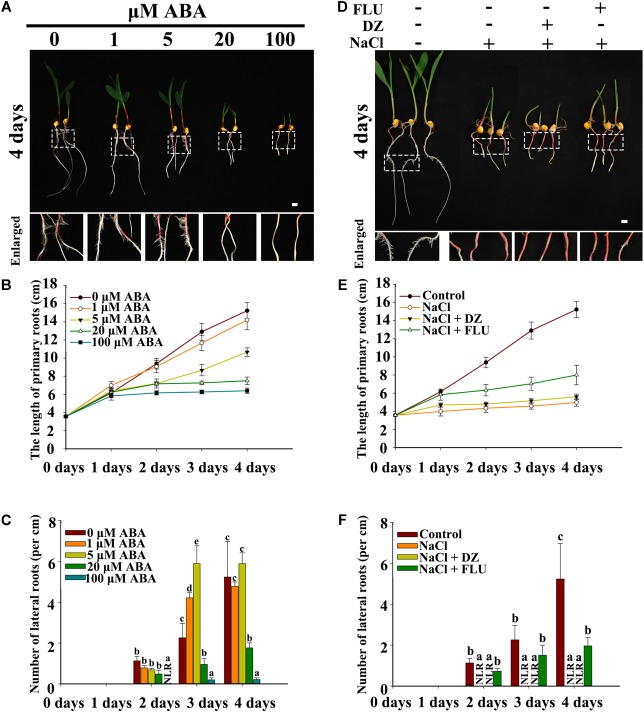
Effects of exogenous ABA and reagents on LR development in maize. **(A)** Phenotypes of wild-type (B73) seedlings under different concentrations of ABA treatments. The white dotted line in the image is the enlarged part. Bars = 1 cm. **(B)** Determination of the PR length in **(A)**. **(C)** Density of the LRs in **(A)**. **(D)** Phenotypes of 3-day-old wild-type (B73) seedlings under different treatments. Treatment concentrations: 200 mM NaCl, 50 μM DZ, and 5 μM FLU. The white dotted line in the image shows the enlarged part. Bars = 1 cm. **(E)** Determination of the PR length in **(D)**. **(F)** Determination of the LR density in **(D)**. The data represent the means ± SEs of five replicates, with 10 seedlings each in **(A–F)**. The different letters represent significant differences between the treatment and the control (*P* < 0.05, based on Student’s *t*-test).

Exogenous ABA, the ABA biosynthesis inhibitor FLU and the ABA catabolism inhibitor DZ were used to measure the function of ABA in NaCl-inhibited LR development. The results indicated that PRs and LRs responded differently to ABA, with PR growth being more sensitive to ABA than was LR emergence. In addition, 1 and 5 μM ABA inhibited PR growth and increased LR emergence (Figure 3A–C), and relatively high concentrations of exogenous ABA (>20 μM) inhibited both PR growth and LR emergence (Figure 3B,C and Supplementary Figurs S4A,B).

**FIGURE 4:**
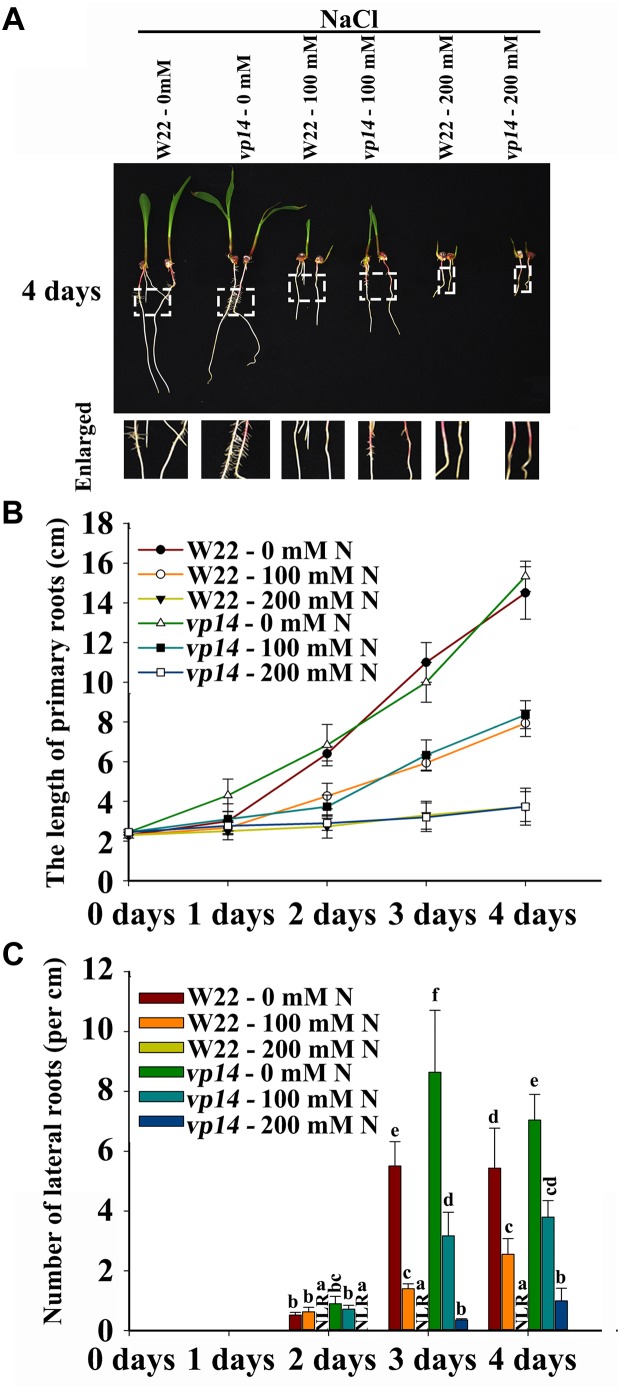
Phenotypes of *vp14* seedlings under salt stress. **(A)** Phenotypes of *vp14* and W22 under salt stress. Bars = 1 cm. **(B)** The length of the PRs in **(A)**. N, NaCl. **(C)** Number of LRs in **(A)**. N, NaCl. The data represent the means ± SEs of five replicates, with 10 seedlings each in **(A–C)**. The different letters represent significant differences (*P* < 0.05, based on Student’s *t*-test).

FLU and DZ were used to inhibit ABA biosynthesis and catabolism, respectively. The results indicated that DZ treatment increased ABA accumulation and that FLU treatment decreased ABA accumulation in the roots of both the maize wild type and ABA biosynthesis mutant *vp14* (Supplementary Figure S5). Moreover, under NaCl treatment, high levels of ABA accumulated, and ABA accumulation increased in response to DZ and decreased in response to FLU (Supplementary Figure S6). Additionally, 200 mM NaCl inhibited PR elongation and LR density, and the inhibition of LR density but not PR growth was dramatically rescued by the ABA biosynthesis inhibitor FLU (Figure 3D–F), while DZ, an ABA catabolism inhibitor, increased the inhibition of LR emergence by NaCl (Figure 3D–F). These results indicated that ABA played an important role in NaCl-regulated LR development. The results also showed that, compared with LRs, PRs may be regulated by NaCl via different signaling pathways. The ABA biosynthesis mutant *vp14* was used to observe the effects of ABA on LR development in response to salt treatments (Tan et al., 1997; Settles et al., 2004), and the results showed that ABA biosynthesis decreased in the mutant in response to the control and NaCl treatments. Two homozygous mutants were selected and used for analyses (Supplementary Figure S5E). We found that the length of the PRs was similar between the *vp14* and wild-type plants; however, the number of LRs of *vp14* was significantly greater than that of the wild type under natural conditions and under the 100 mM NaCl treatment (Figure 4A–C). However, 200 mM NaCl still reduced the length of PRs and the number of LRs to the same degree in both *vp14* and wild-type seedlings (Figure 4A–C and Supplementary Figure S7). These results were in agreement with those in Figure 3, which showed that FLU dramatically rescued the inhibition of LR density, not PR growth, under NaCl treatment. We also found that the quiescence of LRs induced by ABA could be rescued when the maize seedlings were transferred to a new nutrient solution that lacked ABA (Supplementary Figure S8), which is similar to a related situation involving *Arabidopsis* (Duan et al., 2013).

### NaCl Treatment Increases the Auxin Accumulation in Maize Roots

**FIGURE 5:**
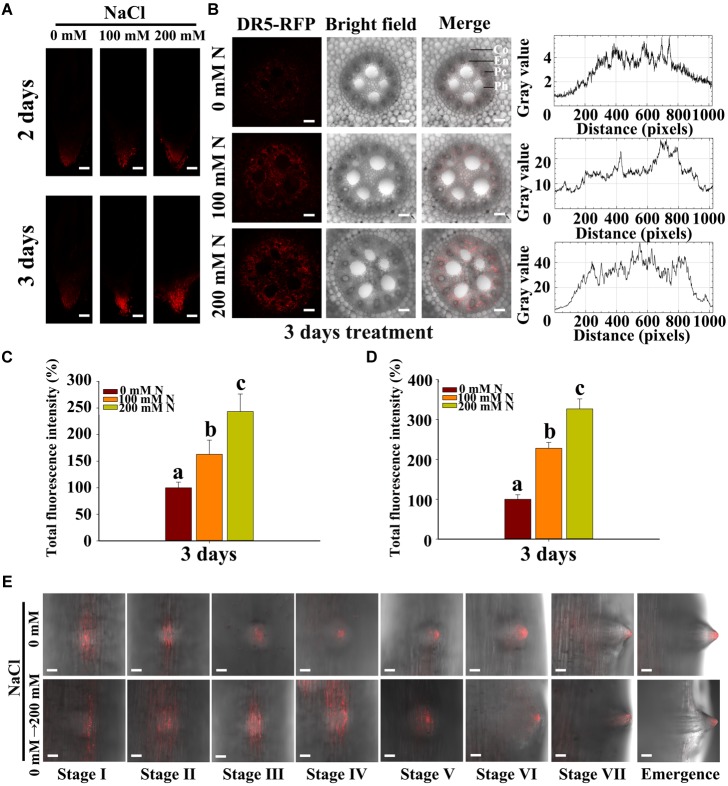
Salt stress stimulated the endogenous auxin response in the root tips and LRs. **(A)** Fluorescence of DR5-GFP in the tips of PRs under 0, 100, or 200 mM NaCl treatment was observed via fluorescence microscopy. Bars = 100 μm. **(B)** Observations of the fluorescence of DR5-GFP in transverse root sections under 0, 100, or 200 mM NaCl treatment via confocal microscopy. The right curve shows the fluorescence intensity of DR5-RFP. Co, cortex; En, endodermis; Pc, pericycle; Ph, Phloem. Bars = 50 μm. N, NaCl. **(C)** Total fluorescence intensity of the fluorescence part in **(A)**. N, NaCl. **(D)** Total fluorescence intensity of the fluorescence part in **(B)** (Student’s *t*-test). N, NaCl. **(E)** Observations via confocal microscopy of the fluorescence of DR5-GFP at different developmental stages of LRs under 0 or 200 mM NaCl treatment for 8 h. Bars = 50 μm. The data represent the means ± SEs of five replicates, with 10 seedlings each. The images were selected from five replicates, with 10 seedlings per replicate. The different letters represent significant differences between the treatment and the control (*P* < 0.05, based on Student’s *t*-test).

Auxin has been reported to be a key signaling hormone involved in LR development, and auxin treatments have been shown to increase LR formation in rice, maize and barley (Casimiro et al., 2003; De Smet et al., 2007; Orman-Ligeza et al., 2013). Therefore, the genes involved in auxin biosynthesis (*ZmTAR1*, *ZmVT2*, and *ZmYUC1* to *ZmYUC8*) (Phillips et al., 2011; Matthes et al., 2018), response (*ZmARF25* and *ZmARF34*) and transport (*ZmPIN1a*, *ZmPIN2*, *ZmPIN10a*, *ZmPIN10b*, and *ZmAUX1*) were analyzed (Paponov et al., 2005; von Behrens et al., 2011; Korasick et al., 2013) with respect to their participation in the salt stress response. The results indicated that the expression of all of these genes was induced by NaCl treatment (Supplementary Figures S9, S10). Because the effects of auxin on plant development are associated with not only its concentration but also its distribution (Shkolnik-Inbar and Bar-Zvi, 2010), *DR5rev::mRFPer* transgenic lines were used to analyze the response maxima and distribution of auxin. By scanning along the root, we found that DR5 accumulated in the root tips and within the LRI zone (Figure 5A,E). NaCl treatment increased the response maxima of auxin in both the root tips and the phloem (Figure 5A–D). The results also showed that DR5 accumulated within the LRP and in the tips of LRs during development (Figure 5E). Moreover, NaCl treatment increased DR5 accumulation and altered the distribution of auxin in the LRI zone at the early stages of LR development (Figure 5E). Under control conditions, DR5 accumulated in the tips of LRs, while NaCl treatment increased the accumulation of DR5 at the base of the LRs but decreased the accumulation in the tips of LRs, especially at the early stages (stage I to stage IV) (Figure 5E) but less so at the late stages (stage VII and stage VIII). Our results also showed that the distribution of auxin did not change in the PRs, although the auxin response maxima were increased by NaCl treatment (Figure 5A).

### IAA Is Involved in NaCl-Regulated LR Development in Maize

**FIGURE 6:**
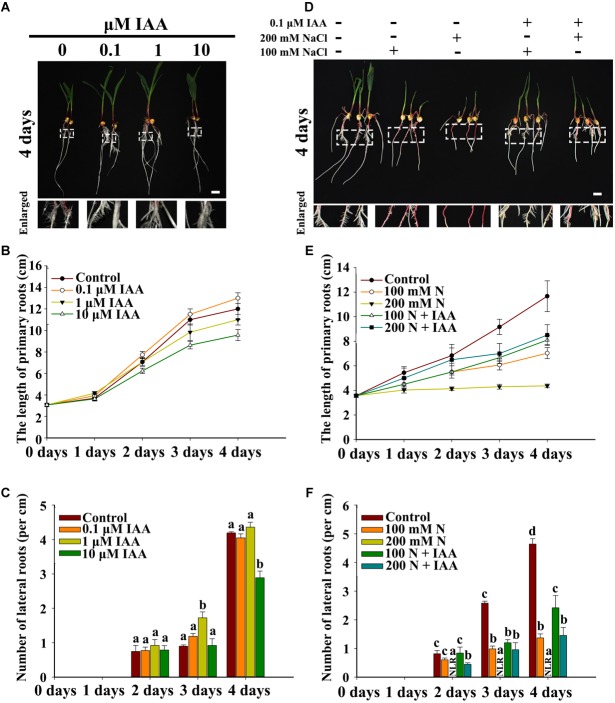
Effects of exogenous IAA on LR development. **(A)** Phenotypes of wild-type (B73) seedlings under different concentrations of IAA. The white dotted line in the image shows the enlarged part. Bars = 1 cm. **(B)** Length of the PRs of the wild-type (B73) seedlings under different concentrations of IAA. **(C)** The LR density of the wild-type (B73) seedlings under different IAA treatments. **(D)** Phenotypes of wild-type (B73) seedlings under different NaCl treatments with or without 1 μM IAA. Bars = 1 cm. **(E)** Determination of the PR length of wild-type (B73) seedlings under the different NaCl treatments in **(D)**. The white dotted line in the image shows the enlarged part. Bars = 1 cm. **(F)** Determination of the LR density of wild-type (B73) seedlings under the different NaCl treatments in **(D)**. N, NaCl. The data represent the means ± SEs of five replicates, with 10 seedlings each in **(B–D)**. The different letters represent significant differences between the treatment and the control (*P* < 0.05, based on Student’s *t*-test).

Exogenous IAA was used to mimic the effects of auxin on PR and LR development. The results indicated that 0.1 μM IAA slightly increased PR elongation but did not significantly affect LR density (Figure 6A–C and Supplementary Figure S11A). Moreover, 10 μM IAA inhibited PR elongation and slightly decreased LR density (Figure 6A–C and Supplementary Figure S11A). NaCl treatment inhibited PR elongation and LR density, and IAA partially rescued the NaCl-induced inhibition of PR elongation and LR density (Figure 6D–F and Supplementary Figure S11B). These results indicated that auxin probably participates in NaCl-regulated LR development.

### ABA Is Involved in NaCl-Regulated Auxin Distribution in Maize

In *Arabidopsis*, NaCl treatment inhibits PR and LR elongation by decreasing auxin accumulations (Liu et al., 2016). In maize, NaCl treatment inhibited PR and LR elongation but increased auxin response maxima (Figure 5, 6). Therefore, the effects of NaCl on root development differ between *Arabidopsis* and maize. Our results showed that both ABA and auxin participated in NaCl-regulated LR development in maize. To elucidate the effects of NaCl on LR development, we measured the fluorescence in *pZmPIN1a*::*ZmPIN1a*:*YFP* and *DR5rev*::*mRFPer* transgenic lines to elucidate how ABA and the auxin response maxima and distributions change in maize roots.

**FIGURE 7:**
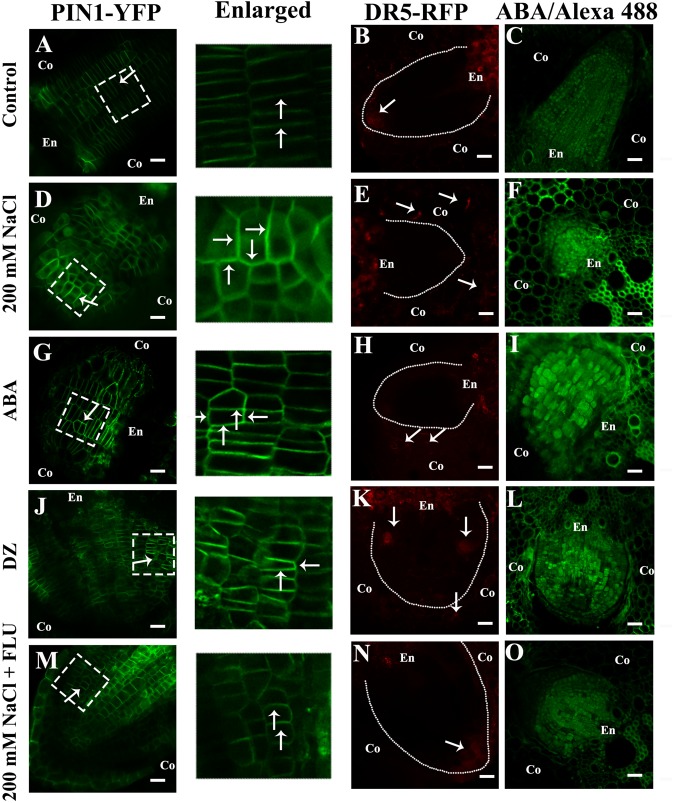
NaCl promoted ABA accumulation and altered the distribution of PIN1 and auxin. **(A–O)** show the transverse sections of the LRs. **(A,D,G,J,M)** show the fluorescence of PIN1-YFP. **(B,E,H,K,N)** show the fluorescence of DR5-RFP. The white dotted lines represent the profile of the LRP, and the arrow points to the distribution of DR5-RFP in the root tips of LRs under different treatments. **(C,F,I,L,O)** show the immunofluorescence of ABA. The white dotted line in images **(A,D,G,J,M)** show the enlarged part. The arrow points to the location of PIN1-YFP in the LRs under different treatment concentrations: 200 mM NaCl, 100 μM ABA, 5 μM DZ, and 10 μM FLU. All the pictured transmission graphs are given in Supplementary Figure S13. The figures were selected from five replicates, with 10 seedlings per replicate, and all of the seedlings presented similar results.

Previous results showed that NaCl treatment increased the accumulation of DR5 (Figure 5). The fluorescence of DR5 was located in the tips of LRs under natural conditions (Figure 7B), but it diminished under the 200 mM NaCl treatment (Figure 7E,H,K and Supplementary Figure S12B). The exogenous ABA and DZ treatments also reduced the accumulation of DR5 in the tips of the LRs (Figure 7H,K and Supplementary Figure S12B). The density of the LRs was also inhibited by the ABA and DZ treatments (Supplementary Figure S12). Similarly, FLU treatment altered the distribution of DR5 under the 200 mM NaCl treatment (Figure 7M and Supplementary Figure S12B). The inhibition of the LR density was also partially rescued by the FLU treatment (Figure 7B,C and Supplementary Figure S12A). The IAA treatment slightly rescued the inhibition of LR density caused by NaCl and ABA, but it did not alter the distribution of DR5 under either treatment (Supplementary Figure S13).

### NaCl Treatment Regulates Auxin Distribution by Affecting the Polar Localization of PIN1

The auxin influx carriers AUX1/LAX and auxin efflux carrier PIN coordinate auxin polar transport together (Adamowski and Friml, 2015; Fabregas et al., 2015). Previous studies have shown that salt stress affects the distribution of PIN1 in *Arabidopsis* roots (Jiang et al., 2016). Therefore, we used a *ZmPIN1a-YFP* transgenic line to elucidate how NaCl affects the polar localization of ZmPin1, and our results showed that NaCl treatment affected the polar localization of auxin (Figure 7A,D and Supplementary Figure S13). The exogenous ABA and DZ treatments altered the accumulation and polar localization of ZmPIN1 (Figure 7G,J and Supplementary Figure S13), respectively. When ABA biosynthesis was inhibited by exogenous FLU, the localization of ZmPIN1 that occurred under NaCl treatment was restored (Figure 7M and Supplementary Figure S13). Exogenous IAA treatment increased ZmPIN1 expression but did not affect its distribution; exogenous IAA plus ABA also increased ZmPIN1 expression and disrupted its distribution (Supplementary Figure S13). The NaCl, ABA and DZ treatments increased the accumulation of ABA in the LRs, while the FLU treatment reduced the ABA accumulation (Figure 7C,F,I,L,O).

**FIGURE 8:**
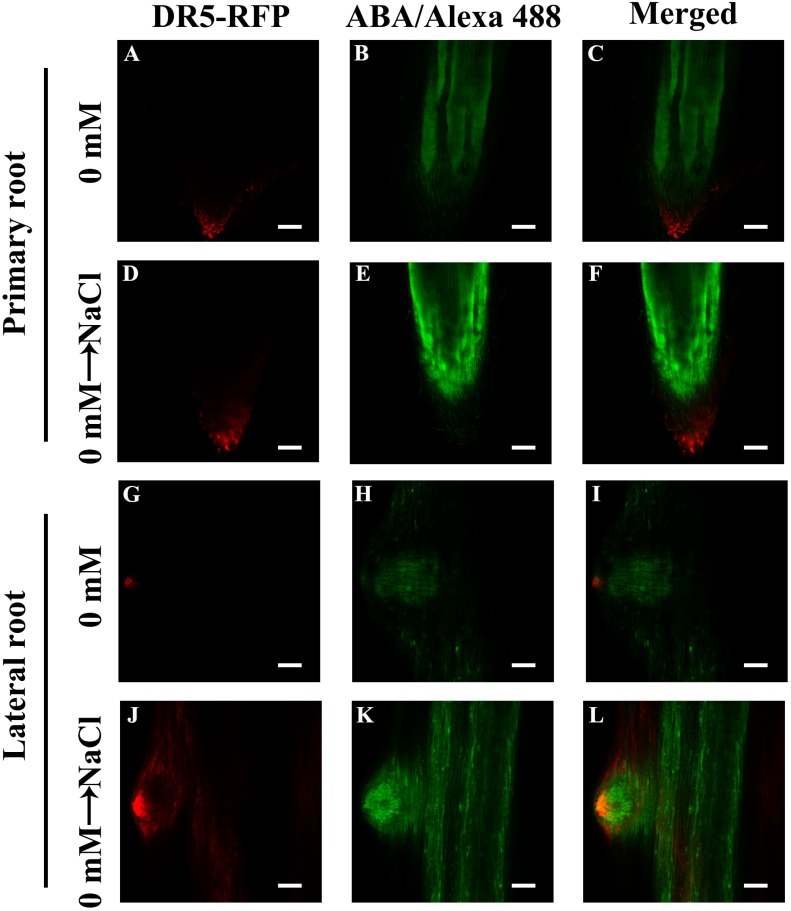
Distribution of ABA and auxin within root tips and LRs. **(A,D,G,J)** indicate the immunofluorescence of DR5-RFP. **(B,E,H,K)** indicate the immunofluorescence of ABA. The immunofluorescence of DR5-RFP was observed in living (plant body) tissue and then fixed to the ABA immunofluorescence observation, superimposing the two images. The roots grew for 3 days, after which 200 mM NaCl treatment was applied for 8 h **(D–F,J–L)**. Bars = 100 μm **(A–F)**. Bars = 50 μm **(G–L)**. The figures were selected from five replicates, with 10 seedlings per replicate, and all of the seedlings presented similar results.

Our results showed that ABA plays a pivotal role in NaCl-regulated ZmPin1 distribution. Therefore, we also measured the ABA distribution under the same treatments. The results showed that ABA accumulated specifically in LRs, excluding the tips of LRs. The NaCl, exogenous ABA and DZ treatments increased the accumulation of ABA, while FLU treatment decreased its accumulation. The exogenous ABA and DZ treatments increased the auxin response maxima, while exogenous IAA treatment did not affect the accumulation of ABA under the control treatment conditions or in response to NaCl treatment (Figure 7A–O and Supplementary Figures S12, S13). On the basis of these results, we hypothesized that the accumulation and distribution of ABA affected the polar localization of ZmPIN1 and auxin. Therefore, we measured the distribution of ABA and auxin together. The results showed that the distribution of ABA and auxin complemented each other in terms of spatial positioning in the PRs and LRs; however, auxin was localized in the root tips (below the quiescent center), while ABA was localized in the PRs and LRs, excluding the root tips (above the quiescent center). NaCl treatment expanded the distribution of ABA to the tips of the LRs, which are regions of auxin distribution, but did not alter the distribution of ABA in the PRs. Therefore, the localization of auxin in the root tips was disrupted in the LRs (Figure 8).

## Discussion

Although the mechanism by which NaCl and other environmental stressors regulate root development has been well investigated in *Arabidopsis* (Deak and Malamy, 2005; Li et al., 2011; Ding et al., 2015), the functions of some hormones, such as auxin, ABA and cytokinin, that are reported to be involved in NaCl-regulated root development (Shkolnik-Inbar and Bar-Zvi, 2010; Orman-Ligeza et al., 2013; Shu et al., 2016) are still unclear in maize. Our data showed that the inhibition of LRI by NaCl required ABA; NaCl treatment increased the accumulation of ABA. The distribution of auxin, regulated by ABA, inhibits LRI and development. In *Arabidopsis*, NaCl treatment increases ABA and decreases auxin accumulation (Duan et al., 2013; Ding et al., 2015), but the response mechanism following salt stress may differ in maize.

### ABA Accumulates in LRs and Is Involved in the NaCl Regulation of LR Development

Abscisic acid is reported to inhibit LR development at the post-emergence stage under different environmental stressors, indicating that LRs are more sensitive than PRs to ABA in *Arabidopsis*, a dicotyledonous species (Signora et al., 2001; De Smet et al., 2003; Duan et al., 2013). However, the effects of ABA on LR development in monocotyledonous plants, such as maize and rice, still need to be studied. In particular, how the localization of ABA affects LR development should be elucidated.

Previous studies have shown that the expression of *ABI1* in the endodermis and pericycle of *Arabidopsis* roots can rescue the inhibition of root development by ABA, while the expression of *ABI1* in other tissues cannot (Duan et al., 2013). These results show that ABA must localize to specific tissues to function. Using immunolocalization methods, we observed the localization of ABA under control and high-NaCl conditions. Our results showed that ABA accumulated in the tips of the roots above the quiescent center in LRs and, to a lesser degree, in LRP of maize (Figure 2A,E). ABA biosynthesis, ABA catabolism and expression of response genes increased under NaCl treatment (Supplementary Figure S2); these results are similar to those in *Arabidopsis* (Ondzighi-Assoume et al., 2016). By observing transverse root sections, we found that ABA accumulated in the endodermis, pericycle, and phloem under control conditions (Figure 3B), which is different from the results in *Arabidopsis*; in *Arabidopsis*, ABA accumulated in the endodermis (Ondzighi-Assoume et al., 2016). This difference may be related to the differences in root structure between monocots and dicots. In maize, LRP originate in the pericycle (Orman-Ligeza et al., 2013) and represent the primary location of ABA accumulation. Therefore, these results indicated that ABA participated in the regulation of LR development. The NaCl and exogenous ABA treatments increased the accumulation of ABA in the PRs and LRs and caused its distribution to expand to all tissues. A similar result was also reported in *Arabidopsis* in response to KNO_3_ treatment (Ondzighi-Assoume et al., 2016).

ABA has been indicated to mediate osmotic stress- and NaCl stress-dependent LR inhibition in *Arabidopsis* (De Smet et al., 2003; Deak and Malamy, 2005; He et al., 2005; Duan et al., 2013). Our results showed that the inhibition of LRs by NaCl was restored by the exogenous ABA biosynthesis inhibitor FLU in maize and was weakened in the ABA biosynthesis mutant *vp14* (Figure 3D–F, 4). These results indicated that ABA also plays an important role in NaCl-inhibited LR development in maize. Previous studies have indicated that NaCl treatment induces the quiescence of LRs in *Arabidopsis* (Duan et al., 2013). Our results also showed that ABA could induce the quiescence of LRs in maize, and the quiescence of LRs induced by ABA could be rescued when the maize seedlings were transferred to a new nutrient solution that lacked ABA (Supplementary Figure S8). These results showed that ABA might play similar roles in regulating the quiescence of LRs in both maize and *Arabidopsis*.

### NaCl Enhances Auxin Response Maxima and Inhibits Root Development in Maize

Auxin is reported to act as a key regulator that is involved in all stages of LR development (Lavenus et al., 2013). Environmental stressors such as NaCl, osmotic stress and exogenous ABA reduce auxin response maxima and inhibit PR and LR growth in *Arabidopsis* (De Smet et al., 2003; Ding et al., 2015). We found different results when we treated maize with NaCl: the auxin response maxima in the roots increased under both the 100 and 200 mM NaCl treatments (Figure 5). When *DR5rev::mRFPer* transgenic lines were used to detect fluorescence in the PRs, auxin was distributed primarily in the root tips (below the quiescent center), and the NaCl and exogenous ABA treatments increased the auxin response maxima. Additionally, these NaCl treatments inhibited LRI and growth (Figure 1, 7 and Supplementary Figure S1). We also measured the expression of genes related to auxin pathways in response to these treatments and found that the NaCl and ABA treatments increased the expression of genes involved in auxin biosynthesis, auxin catabolism and the auxin response in maize (Supplementary Figure S9). Moreover, our results showed that the ABA biosynthesis inhibitor FLU partially rescued the NaCl-induced inhibition of LR development (Figure 7).

These results indicated that the auxin response maxima increased under NaCl treatment, and this increase correlated with an increase in ABA concentrations. These results are not consistent with processes in *Arabidopsis* because, in that species, an increase in ABA was shown to reduce the response maxima of auxin and inhibit LR growth (Shkolnik-Inbar and Bar-Zvi, 2010; Duan et al., 2013). Auxin plays a central role during all stages of LR development, and a decrease in auxin biosynthesis and response affects LRI and growth in *Arabidopsis* (Lavenus et al., 2013). Therefore, it is very interesting that NaCl increased ABA and the auxin response maxima in maize and that this increase did not affect LRI but inhibited LR growth (Figure 2, 6, 7). To better understand these effects, we used different concentrations of exogenous IAA to treat maize; the results showed that 0.1 μM IAA increased PR elongation, while 1–10 μM IAA slightly inhibited PR growth (Figure 6), and only 10 μM IAA inhibited LR growth. We also determined the auxin response maxima in the LRs in response to treatment with exogenous ABA as well as FLU and DZ, which are inhibitors of ABA biosynthesis and ABA catabolism, respectively. The results indicated that exogenous ABA and DZ slightly increased the auxin response maxima in LRs under NaCl treatment, while FLU slightly decreased the maxima (Figure 8). In addition, ABA and DZ inhibited LR development, while FLU rescued the NaCl-induced inhibition of LR development; however, the results also showed that exogenous IAA could rescue the NaCl-induced inhibition of LRs (Supplementary Figures S12, S13). These results indicated that the auxin response maxima may not be directly correlated with the phenotype. Therefore, we suggest that, in maize, it may be not the concentration but the distribution of auxin that regulates LR development under NaCl stress.

### ABA Participates in NaCl-Regulated LR Development by Regulating the Distribution of Auxin

Auxin affects plants not only by its concentration but also by its distribution (De Smet et al., 2007; Adamowski and Friml, 2015; Xi et al., 2016). An altered auxin distribution can result in severe phenotypes, such as that of the *pin1* mutant, which is characterized as having a stem that is nearly devoid of organs such as leaves and flowers (Okada et al., 1991). Polar auxin transport also plays a central role in root development, as it affects LR formation and root gravitropism (Shkolnik-Inbar and Bar-Zvi, 2010; Xi et al., 2016). Compared with wild-type plants, *pin* mutants progress more slowly through development, and LRs are generated at a lower frequency or cannot be formed at all (Benkova et al., 2003; Adamowski and Friml, 2015). Therefore, we also measured the auxin distribution in response to different treatments and found that auxin was located in the LRP and in the tips of both PRs and LRs (Figure 5A,E), which is complementary to the distribution of ABA under control conditions (Figure 8). NaCl treatment did not alter the distribution of auxin in the PRs but disrupted the distribution of auxin in the LRs. In response to NaCl treatment, the location of the auxin changed from being in the LR tips to having an unorganized distribution, and the auxin response maxima increased (Figure 6, 8 and Supplementary Figure S13). The inhibitor of ABA biosynthesis reduced the concentration of ABA in both the control and NaCl treatment groups (Figure 7 and Supplementary Figure S4), and it rescued the NaCl-induced inhibition of LR development and the effects of auxin distribution caused by the NaCl treatments. The FLU treatment restored the unorganized distribution of auxin in the root tips in response to the NaCl treatments (Figure 7B,I and Supplementary Figure S13). The results also showed that the polar growth of the LRs diminished under NaCl treatment; the LRs could initiate formation of LRP, but the LRP grew without polarity and formed swollen tissue, which was similar to results obtained from the ABA and DZ treatments. The FLU treatment rescued this phenotype (Figure 7B,C). 1-N-Naphthylphthalamic acid (NPA) is a chemical compound that inhibits the polar transport of auxin. NPA exhibited a weak inhibitory effect on the development of PRs (Supplementary Figures S14A,B) but significantly inhibited the number of LRs (Supplementary Figures S14C,D). These results show that auxin polar transport is important for LR development. Compared with NaCl, NPA mainly inhibits LR development, which is consistent with the NaCl-based inhibition of LR growth. These results supported our hypothesis: NaCl treatment increased the accumulation of ABA, which disrupted the distribution of PIN1, after which the unorganized distribution of auxin inhibited the polar growth of the LRs.

As previously reported, the polar localization of auxin is correlated with the polarized distribution of auxin transporters (Robert et al., 2013; Adamowski and Friml, 2015). Thus, using the transgenic line *pZmPIN1a*::*ZmPIN1a*:*YFP* under different treatments, we investigated the distribution of PIN1, and the results showed that the polar localization of PIN1 was disrupted by the NaCl and exogenous ABA and DZ treatments (Figure 7 and Supplementary Figure S13). However, the FLU treatment did not alter the PIN1 distribution under control conditions but did rescue the unorganized PIN1 distribution when induced by NaCl. Moreover, exogenous IAA did not alter the distribution of PIN1 under control conditions or NaCl treatment and could not rescue the phenotype induced by NaCl (Figure 7 and Supplementary Figure S13). These results indicated that ABA could affect auxin distribution by altering the localization of PIN1 and that NaCl regulated auxin distribution by promoting the accumulation of ABA in maize LRs.

Previous results have also shown that *ABI1* is expressed specifically in the endodermis and pericycle, both of which constitute the primary location of ABA in our results (Figure 2B), and that *ABI1* rescued the inhibition of LR development by ABA (Duan et al., 2013). High expression levels of *ABI4* were detected in mature regions of PRs and LRs, but relatively low levels were detected in young LRs; no expression was detected in LRP. ABI affects auxin distribution by affecting PIN1 distribution in *Arabidopsis* (Shkolnik-Inbar and Bar-Zvi, 2010), and our results also showed that ABA accumulated in PRs and LRs but, to a lesser extent, in young LRs and LRP (Figure 2). NaCl treatments also altered the complementary distribution of ABA and auxin in LRs; the distribution of ABA and auxin expanded its their original location to encompass the entire LRs (Figure 7, 8). Surprisingly, the complementary distribution of ABA and auxin in the PRs did not change under NaCl treatment. We also found that PRs were less sensitive than LRs to NaCl treatment (Figure 1). These results showed that NaCl induced inhibition via different mechanisms in PRs and LRs in maize.

**FIGURE 9:**
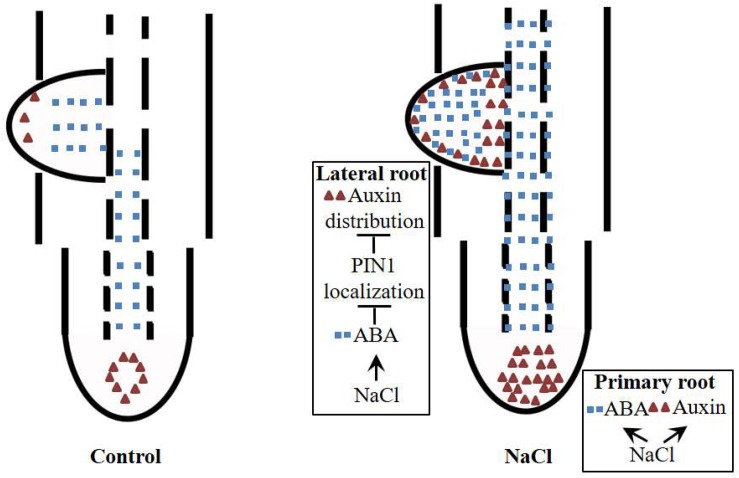
Plausible model for NaCl-regulated LR and PR development. ABA and auxin accumulate in PRs and LRs under NaCl treatment. In PRs, ABA and auxin are induced by NaCl, but the polarity of the distribution does not change. In LRs, ABA is distributed to the top of the LRs, which affects the polarity of the distribution of auxin in LRP by altering the polarity of the localization of PIN1; this phenomenon leads to auxin accumulation around the apices of LRs, resulting in loss of polar growth of LRP. The growth of LRP is ultimately inhibited, and the number of LRs decreases.

Our data suggest that ABA regulates NaCl-modulated LR development and that this regulation occurs by affecting the auxin distribution rather than auxin response maxima. ABA disrupts the polar localization of PIN1, an important auxin transporter (Figure 9). NaCl induced both ABA accumulation and auxin response maxima in the roots (Figure 2, 5); the ABA distribution within the PRs did not change under NaCl treatment, so the localization of auxin in the root tips was preserved, while in LRs, the ABA distribution expanded from the root tips throughout the LRs, which led to an unorganized distribution of auxin and inhibited LR polar growth (Figure 8). PIN1 played an important role in auxin polar transport, and its distribution was affected by NaCl and ABA; this disrupted distribution led to an unorganized distribution of auxin under NaCl treatment and exogenous ABA and DZ treatments (Figure 7). In brief, using the latest immunofluorescence technique and *pZmPIN1a*::*ZmPIN1a*:*YFP* and *DR5rev*::*mRFPer* transgenic lines, we described for the first time the distribution of ABA and auxin under both control conditions and NaCl stress and revealed a novel mechanism by which LR development is regulated in maize.

## Data Availability

All datasets for this study are included in the manuscript and/or the Supplementary Files.

## Author Contributions

Y-GL designed the experiments. CL, M-XC, RL, LZ, XH, SL, XD, YJ, and JX performed the experiments. CL, M-XC, RL, and LZ analyzed the data. CL and Y-GL wrote the manuscript. M-XC, JZ, XZ, and Y-GL critically commented and revised the manuscript.

## Conflict of Interest Statement

The authors declare that the research was conducted in the absence of any commercial or financial relationships that could be construed as a potential conflict of interest.

## Footnotes

1 https://www.antibodies-online.com/

2 http://rsb.info.nih.gov/ij/

3 https://www.maizegdb.org/

4 https://www.ncbi.nlm.nih.gov/genbank/
