# Supplementary material for: Abscisic Acid Regulates Auxin Distribution to Mediate Maize Lateral Root Development Under Salt Stress
Source: Front Plant Sci. 2019 Jun 5;10:716. doi: 10.3389/fpls.2019.00716 (PMC6560076; doi:10.3389/fpls.2019.00716)
Supplement: Supplementary file 1 [file Table_1.docx]

**Table S1** Primers used in this study.

| For qRT-PCR |  |
| --- | --- |
| Actin1F (J01238) | GATTCCTGGGATTGCCGAT |
| Actin1R (J01238) | TCTGCTGCTGAAAAGTGCTGAG |
| GRP2F (GRMZM2G080603T01) | TTCGTTCGTGTCGTGTTTGT |
| GRP2R (GRMZM2G080603T01) | GTCTGCTGAAACCGAGGATG |
| AO1F (GRMZM2G141535) | AACTGCGAGGAGCGATGAAC |
| AO1R (GRMZM2G141535) | CGGTAGTGCGTGGGAGATGT |
| VP14F (GRMZM2G014392) | TTTCTCCCTTTGGATGACAT |
| VP14R (GRMZM2G014392) | CAAGTAACAGCAACCAAGAT |
| ZEPF (GRMZM2G127139) | TCCCTATCATTGTCCTCTCCA |
| ZEPR (GRMZM2G127139) | CTACCTTCATTGTCGGTAATC |
| ABI1F(GRMZM2G300125) | TTGTATGTGGATGCCTGGAT |
| ABI1R(GRMZM2G300125) | TTGATGAGTTGCTGCTGAAG |
| ABI2F(GRMZM2G018485) | CACATTCGTCAGTGGATCTC |
| ABI2R(GRMZM2G018485) | ATTTTCCTTTTATACTCTCCCGAT |
| ABI3F(GRMZM2G133398) | TTCATGTTCGCTGAAGACACG |
| ABI3R(GRMZM2G133398) | ACGAGGAGTTGGACGAGAAG |
| ABI4F(GRMZM2G098063) | GGAGATGCTTCCATGACTTTT |
| ABI4R(GRMZM2G098063) | GTTTCTGGTCTTCTGGAGATAA |
| ABI5(GRMZM2G320754) | GTCTCGGATGAAGGTTCTAAT |
| ABI5(GRMZM2G320754) | TCTCTTAACTCCACCACTACTA |
| ABH1F (GRMZM2G179147) | CCAAACCAAAGCCAACAACTT |
| ABH1R (GRMZM2G179147) | TCGCGGCTCCTATAGTAGTAG |
| ABH4F (GRMZM2G065928) | AAATTGCGTTGGAGGAGAAGA |
| ABH4R (GRMZM2G065928) | CACCATTATCGTAGCGGTGAA |
| TAR1F (GRMZM2G127160) | GTCGCTCCATTCACAGTACTT |
| TAR1R (GRMZM2G127160) | TTGCGTTGCTTCGACGATATC |
| YUCCA1F(GRMZM2G091819) | ATCTCCTGACCACCAACATCTT |
| YUCCA1R(GRMZM2G091819) | TAGCACCTTCTCCGTCATCTCT |
| YUCCA2F(GRMZM2G159393) | TTAGTTAGTCAGTCCAATCCC |
| YUCCA2R(GRMZM2G159393) | AACTCACTCTCACCGTATTACT |
| YUCCA3F(GRMZM2G107761) | ATACATGCCAGTTACAATAG |
| YUCCA3R(GRMZM2G107761) | CCAACATCAATTACAGAAGA |
| YUCCA4F(GRMZM2G141383) | ACCAACACAACAACATCT |
| YUCCA4R(GRMZM2G141383) | TAGAAGAATGAGGAGGAGAA |
| YUCCA5F(GRMZM2G132489) | ATCGTCGTGATGTCTATGTAT |
| YUCCA5R(GRMZM2G132489) | CGCAACTTAATTAGCTTGGA |
| YUCCA6F(GRMZM2G019515) | GGATACTGCTCGGCTTGG |
| YUCCA6R(GRMZM2G019515) | TCTTCCTGATACCTGTCTTACG |
| YUCCA7F(GRMZM2G480386) | AAGAGATGATGAACAACTGAAGA |
| YUCCA7R(GRMZM2G480386) | GCAAGATACGTGGAGTTACC |
| YUCCA8F(GRMZM2G017193) | CCACCACAATCATACCTA |
| YUCCA8R(GRMZM2G017193) | ATCTCTTCAACCTCACAA |
| ZmVT2(GRMZM2G127308) | AGAGGCGCTTGAACGAAGATG |
| ZmVT2(GRMZM2G127308) | GGTACGTCCGGGTCAGCAT |
| PIN1aF (GRMZM2G098643) | ATTTGGTCGTTAATCCTTCAG |
| PIN1aR (GRMZM2G098643) | ACATCCGCTGGCTTTATTCTA |
| PIN2F (GRMZM2G074267) | CTGCTACGACATTTGCTTGG |
| PIN2R (GRMZM2G074267) | CATACAATCCTCACACCTACTTCT |
| PIN10aF (GRMZM2G126260) | CATCCTGAGCACTATGGTAAT |
| PIN10aR (GRMZM2G126260) | AGCTACACTACACTGGCTTCA |
| PIN10bF (GRMZM2G160496) | CCTCACCTGGTCTCTCATCTC |
| PIN10bR (GRMZM2G160496) | AGTATCGTGATGGAGTTCTTC |
| ARF25F (GRMZM2G317900) | GGGCACCAAACCTCAAGAACAGACT |
| ARF25R (GRMZM2G317900) | TGGTGGTATGTGATGTGTCAGTGAA |
| ARF34F (GRMZM2G317900) | ATACAAGGTAGTAAGGAAACGG |
| ARF34R (GRMZM2G317900) | TAAGCAAAAGGAGGTAGCAGAT |
|  | |
| For VP14 mutant |  |
| TIR6 | AGAGAAGCCAACGCCAWCGCCTCYATTTCGTC |
| TIR4 | GCCAWCGCCTCYATTTCGTCGAATCC |
| TIR8.1 | CGCCTCCATTTCGTCGAATCCCCTS |
| vp14-F (GRMZM2G014392) | GCCAAGCCAGCAACACCTCA |
| vp14-R (GRMZM2G014392) | CGCCGACTCCATCTTCAACG |
| vp14-R2 (GRMZM2G014392) | GCCCACCTCTGTTTCCATAC |
