# Supplementary material for: Abscisic Acid Regulates Auxin Distribution to Mediate Maize Lateral Root Development Under Salt Stress
Source: Front Plant Sci. 2019 Jun 5;10:716. doi: 10.3389/fpls.2019.00716 (PMC6560076; doi:10.3389/fpls.2019.00716)
Supplement: Supplementary file 2 [file Image_1.pdf]

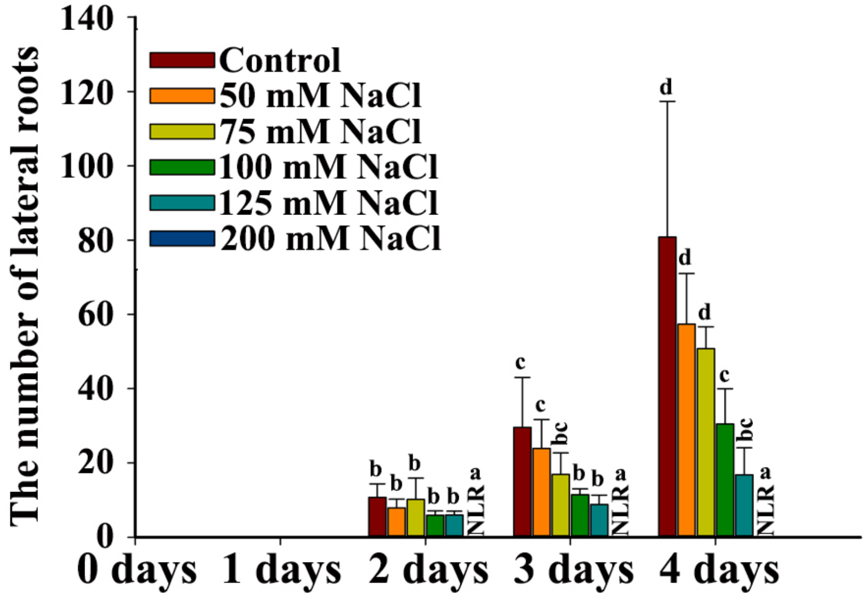

Figure S1 The number of LRs is affected by NaCl treatment.

0 - 4 days after seed imbibition in the absence or presence of 50 mM, 75 mM, 100 mM, 125 mM, 200 mM NaCl. Data represent the means ± SEs of five replicates, with 10 seedlings each treatment. Different letters represent significantly differences between treatment and control ($P < 0.05$, based on Student's t test).

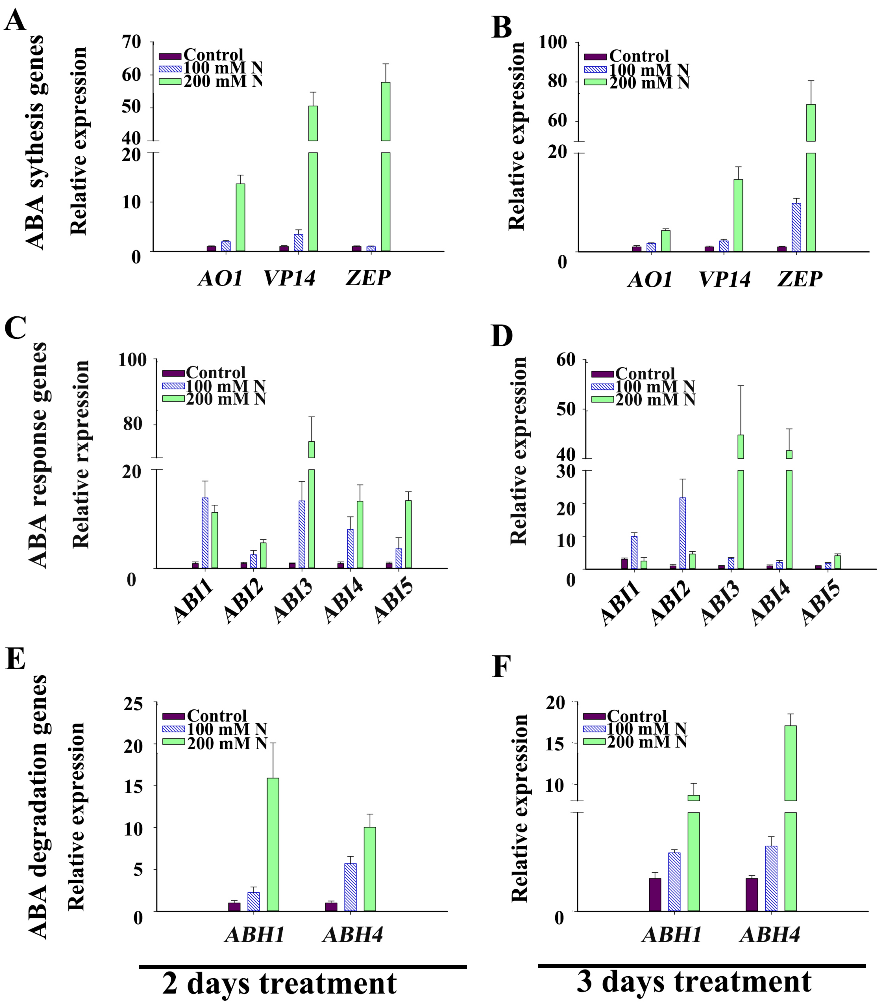

Figure S2 qRT-PCR profiles ABA biosynthesis-, response- and degradation genes in response to NaCl treatment.

The three days seedlings of B73 were used to do the treatments, the seedlings were treated with 100 mM or 200 mM NaCl. Two days and three days roots from treated seedlings were used to extract RNA and do qRT-PCR analysis.

(A) to (B) The relative expression levels of ABA biosynthesis genes, (C) to (D) ABA responsive genes, (E) to (F) ABA degradation genes were analyzed by qRT-PCR under different treatments. Data represent the means ± SEs of three replicates. N = NaCl.

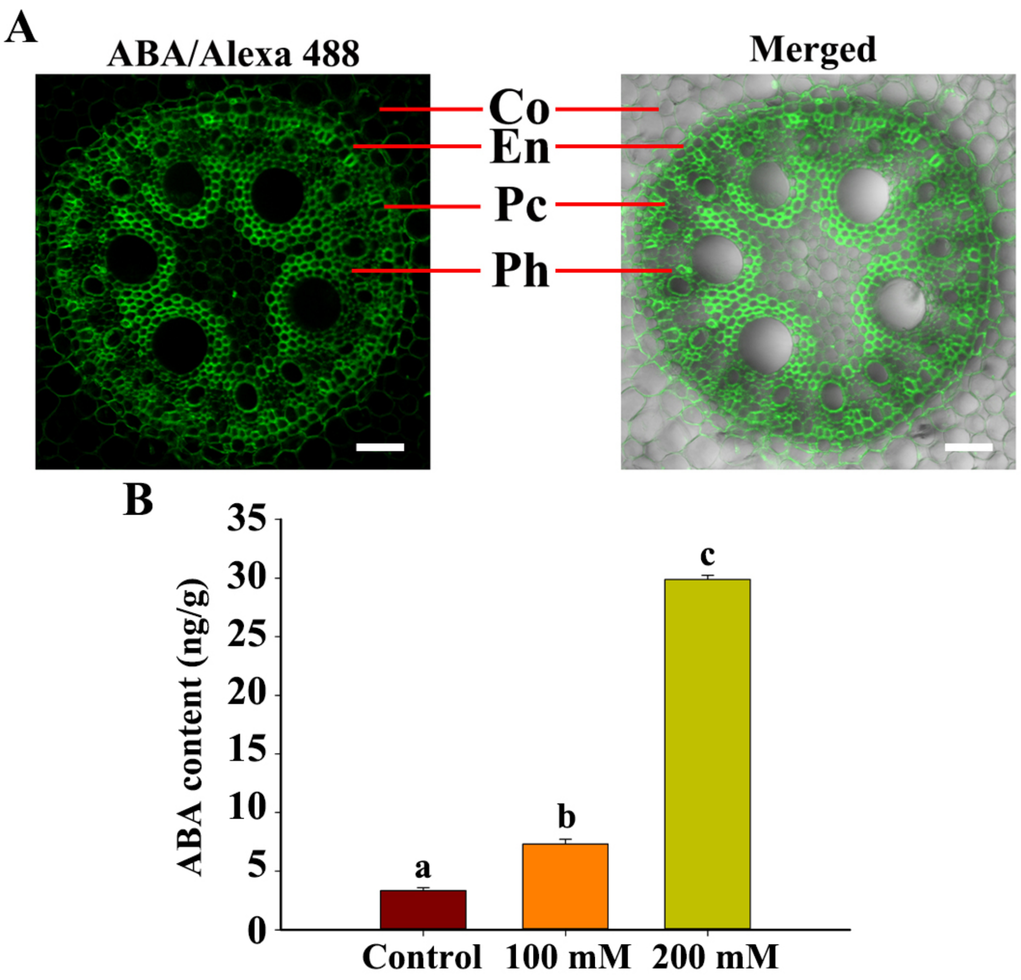

Figure S3 Distribution of ABA in root transection.

(A) ABA is mainly distributed within the endothelium. Co，cortex； En，endodermis; Pc, pericycle; Ph, phloem. Bars = 50 μm.

(B) LC-MS for determination of ABA content. NaCl treatment concentrations: 100 mM and 200 mM.

Data represent the means ± SEs of three replicates, with 10 seedlings each in A, and B. Different letters represent significantly differences between treatment and control ($P < 0.05$, based on Student's t test).

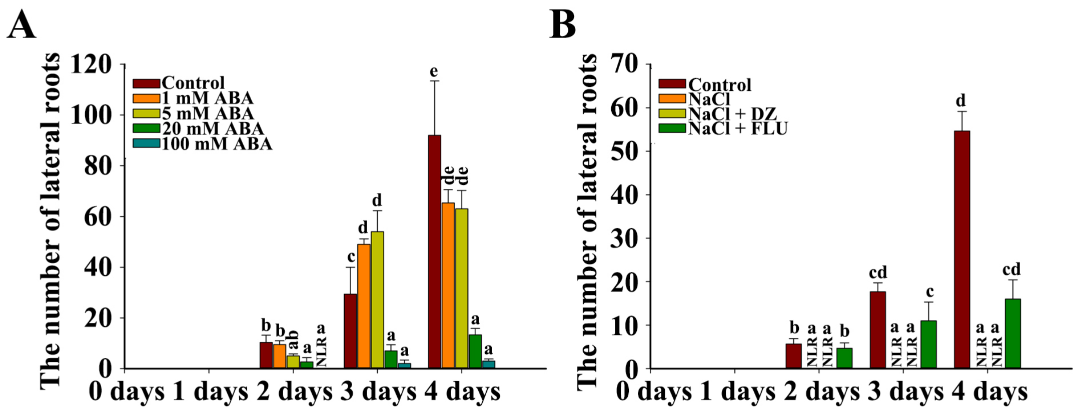

Figure S4 ABA involves in NaCl regulated lateral root development.

(A) The number of lateral roots under gradient concentration ABA. ABA treatment concentrations: 1 μM, 5 μM, 20 μM, 100 μM.

(B) The number of lateral roots under different treatment. Treatment concentrations: 200 mM NaCl, 200 mM NaCl + 5 μM DZ, 200 mM NaCl + 5 μM FLU.

Data represent the means ± SEs of five replicates, with 10 seedlings each in A, and B. Different letters represent significantly differences between treatment and control ($P < 0.05$, based on Student's t test).

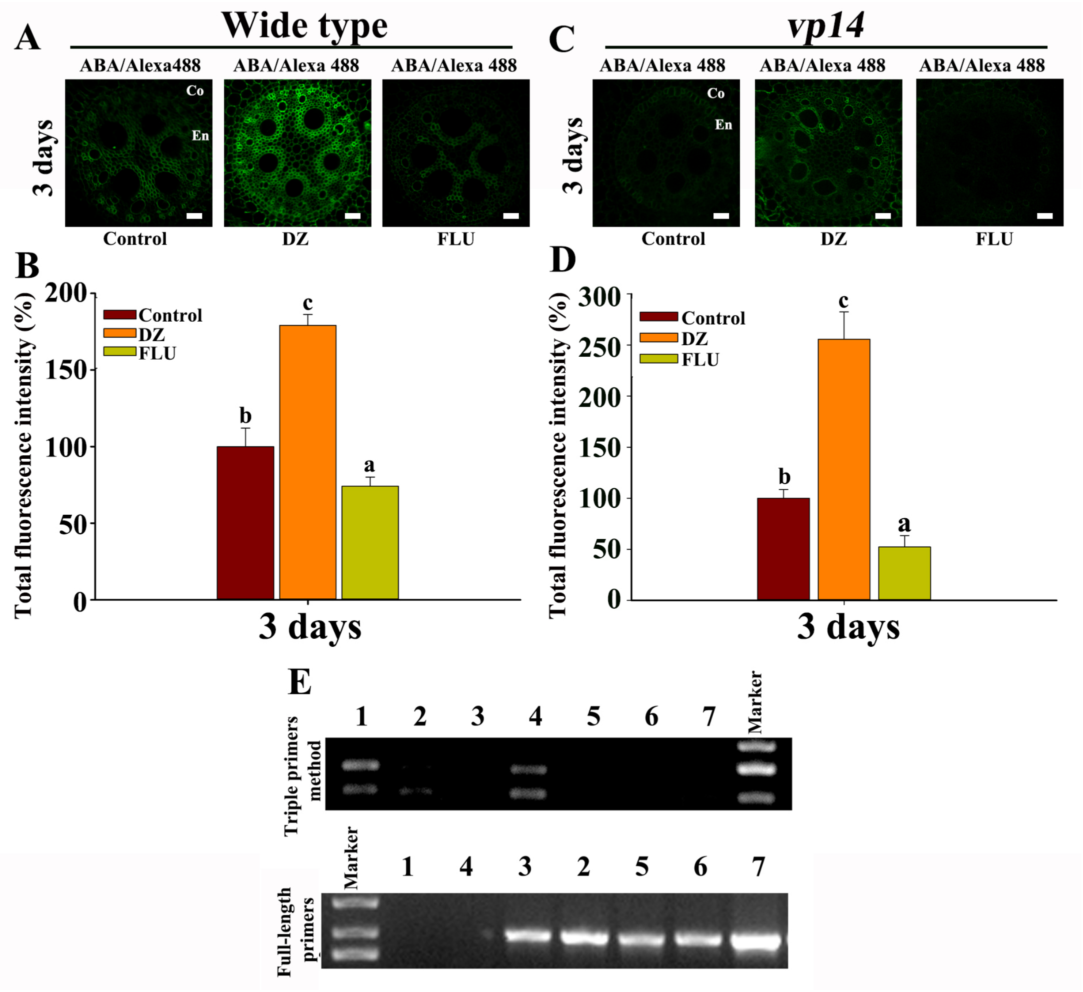

Figure S5 ABA accumulation was largely enhanced by DZ and decreased by FLU obviously in root transection.

(A) and (B) are wide type，(C) and (D) are ABA biosynthesis mutant *vp14.*

(A) and (C) Fluorescence microscope showing ABA accumulation in 3-days-old primary root tips in the Hoagland's Complete Nutrient solution with 0, 5 μM DZ (Diniconazole) or 5 μM FLU (Fluridone). Bars = 50 μm.

(B) and (D) Total fluorescence intensity of the part of the fluorescence in picture (A) and (C), Data represent the means ± SEs of three replicates, with 10 seedlings each in time. Different letters represent significantly differences between treatment and control ($P < 0.05$, based on Student's t test). Photos are selected from five replicates, with 10 seedlings each time.

(E) PCR amplification of double downstream primers showed that 2 and 5 had

insertion sites (Upper), PCR amplification showed that the upstream and downstream primers could not amplify the fragment (Down). Number 1 to 7 represent the PCR results for individual line of *vp14* mutant.

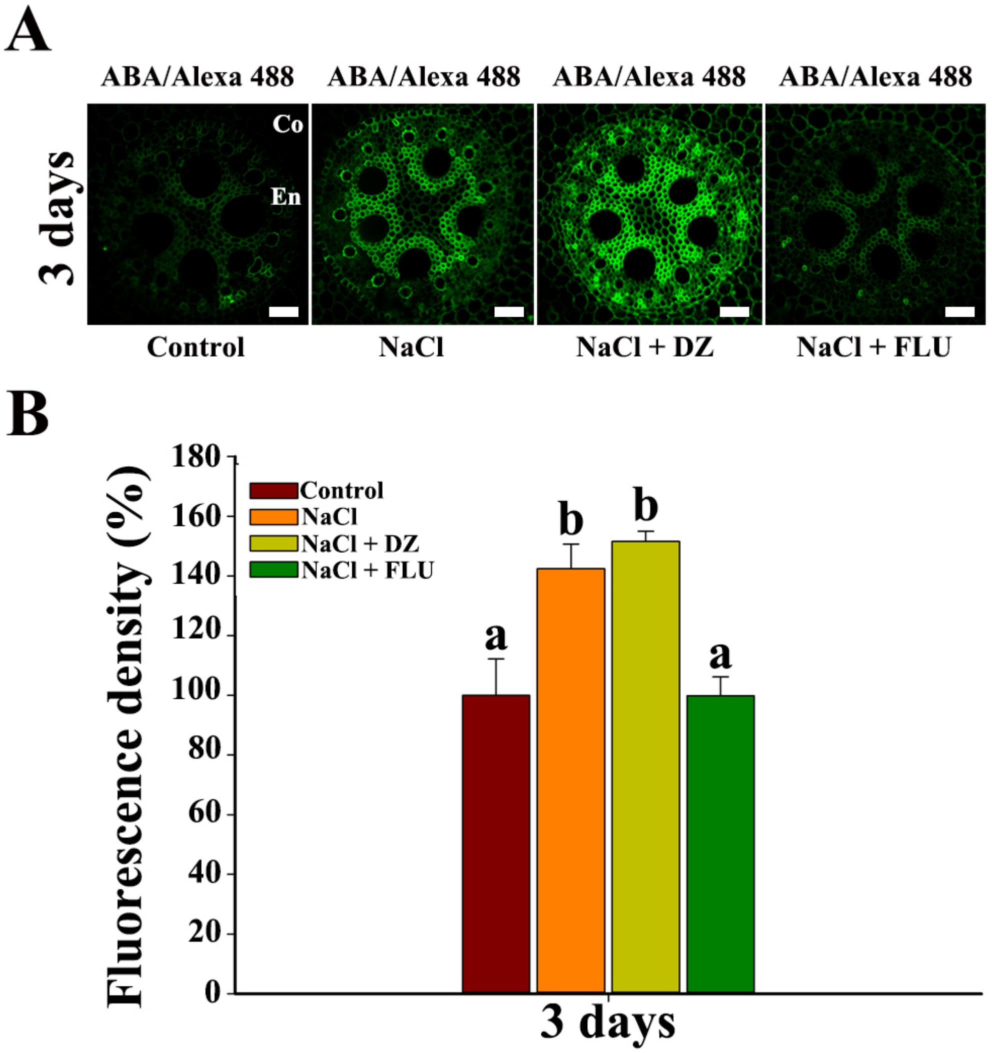

Figure S6 Fluorescence density of ABA under different treatments with transverse root section.

(A) Fluorescence microscope showing ABA accumulation in 3-days-old primary root tips in the Hoagland's Complete Nutrient solution with 0 mM, 200 mM NaCl, 200 mM NaCl + 5 μM DZ, 200 mM NaCl + 5 μM FLU for 3 days. Bars = 50μm.

(B) Fluorescence density (%) of the part of the fluorescence in picture (A). Data represent the means ± SEs of three replicates, with 10 seedlings each time. Different letters represent significantly differences between treatment and control (*P* < 0.05, based on Student's t test).

Photos are selected from five replicates, with 10 seedlings each time.
