# Supplementary material for: Abscisic Acid Regulates Auxin Distribution to Mediate Maize Lateral Root Development Under Salt Stress
Source: Front Plant Sci. 2019 Jun 5;10:716. doi: 10.3389/fpls.2019.00716 (PMC6560076; doi:10.3389/fpls.2019.00716)
Supplement: Supplementary file 3 [file Image_2.pdf]

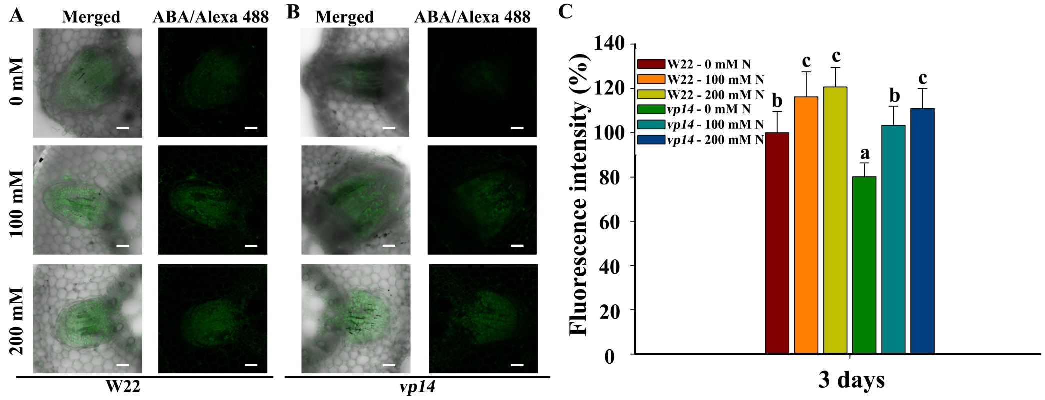

Figure S7 The accumulation of ABA in W22 and *vp14* LRPs.

(A) Fluorescence and fluorescence intensity analysis of ABA in W22 LRPs under Salt Stress. Bars = 50 μm. NaCl concentrations: 0 mM, 100 mM, 200 mM.

(B) Fluorescence and fluorescence intensity analysis of ABA in *vp14* LRPs under Salt Stress. Bars = 50 μm

(C) Fluorescence intensity of figure (A) and (B). Data represent the means ± SEs of five replicates, with 10 seedlings in both A and B. Different letters represent significantly differences ($P < 0.05$, based on Student's t test).

Photos are selected from five replicates, with 10 seedlings each time.

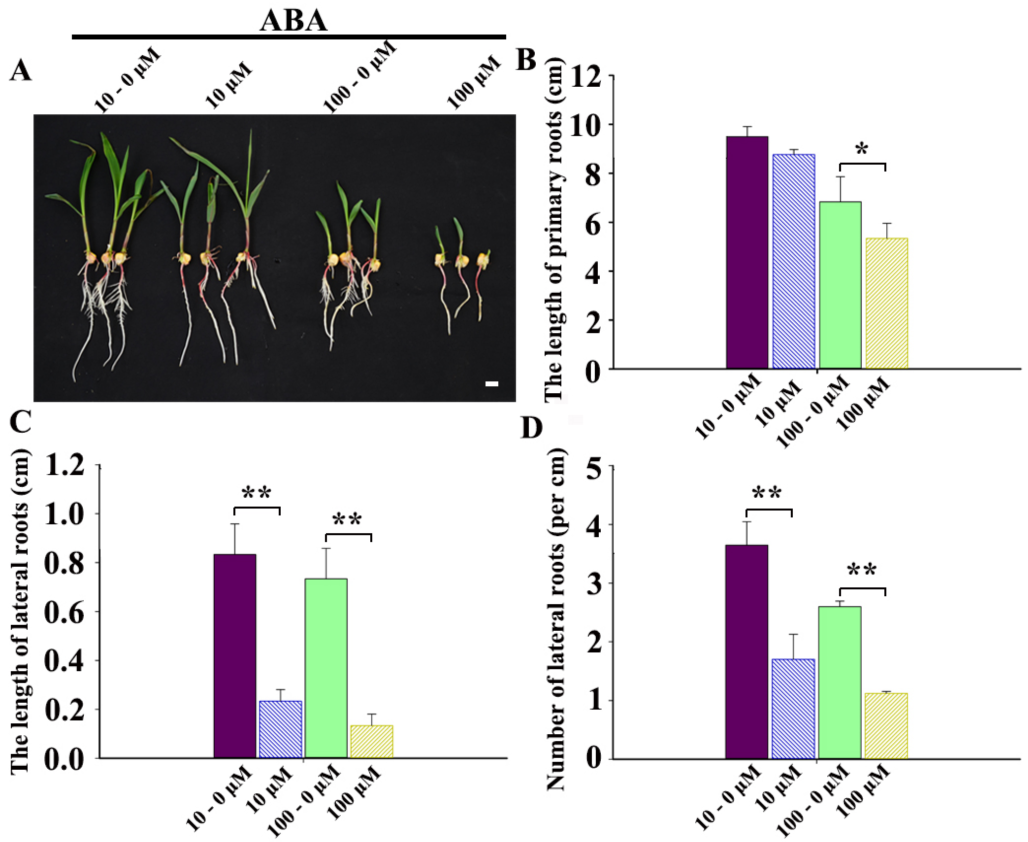

Figure S8 The inhibitory effect of ABA on the development of the LRP is reversible.

(A) The phenotype of wild-type (B73) seedlings after imbibition with 10 - 0 μM,10 μM, 100 - 0 μM, 100 μM ABA. 10 - 0 μM and 100 - 0 μM means seedlings grow in medium with 10 μM /100 μM ABA for 4 days, and then transfer them to the normal medium without ABA for 3 days. 10 μM and 100 μM means seedlings grow in medium with 10 μM /100 μM ABA for 7 days. Bars = 1 cm

(B) The length of primary roots of maize in picture A. Unit = cm

(C) The length of lateral roots of maize in picture A. Unit = cm

(D) Number of lateral roots of maize in picture A per cm. Data represent the means ± SEs of three replicates, with 10 seedlings each time. The asterisks (*) represent significant differences ($P < 0.05$ based on Student's t test).

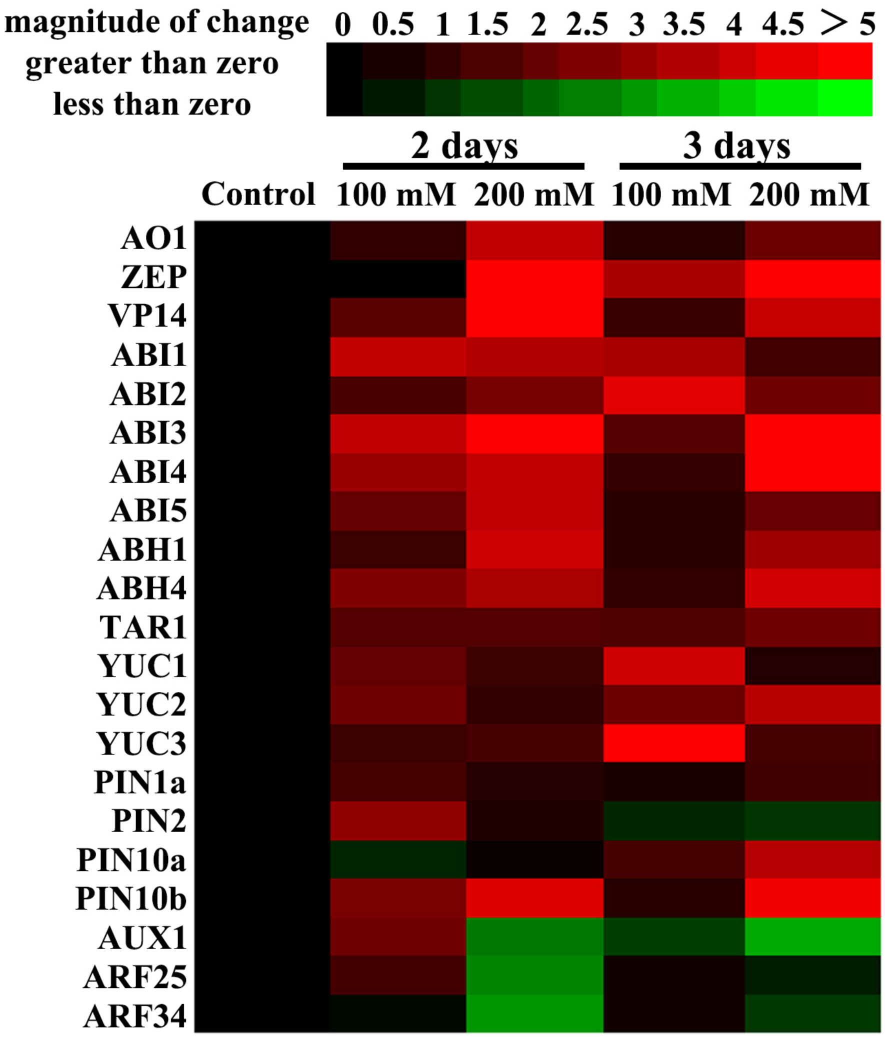

Figure S9 Expression of ABA and auxin related genes under NaCl treatment in maize.

BAR HeatMapper Plus Tool was used to analyze the expression of IAA and ABA related genes after NaCl treatment. NaCl concentrations:100 mM and 200 mM. The relative gene expression values (log2 scale of qRT-PCR, n = 3, technical replicates) were analyzed using the R language programmed heatmap format. Red and green colors represent the up-regulation or down-regulation of gene expression, respectively.

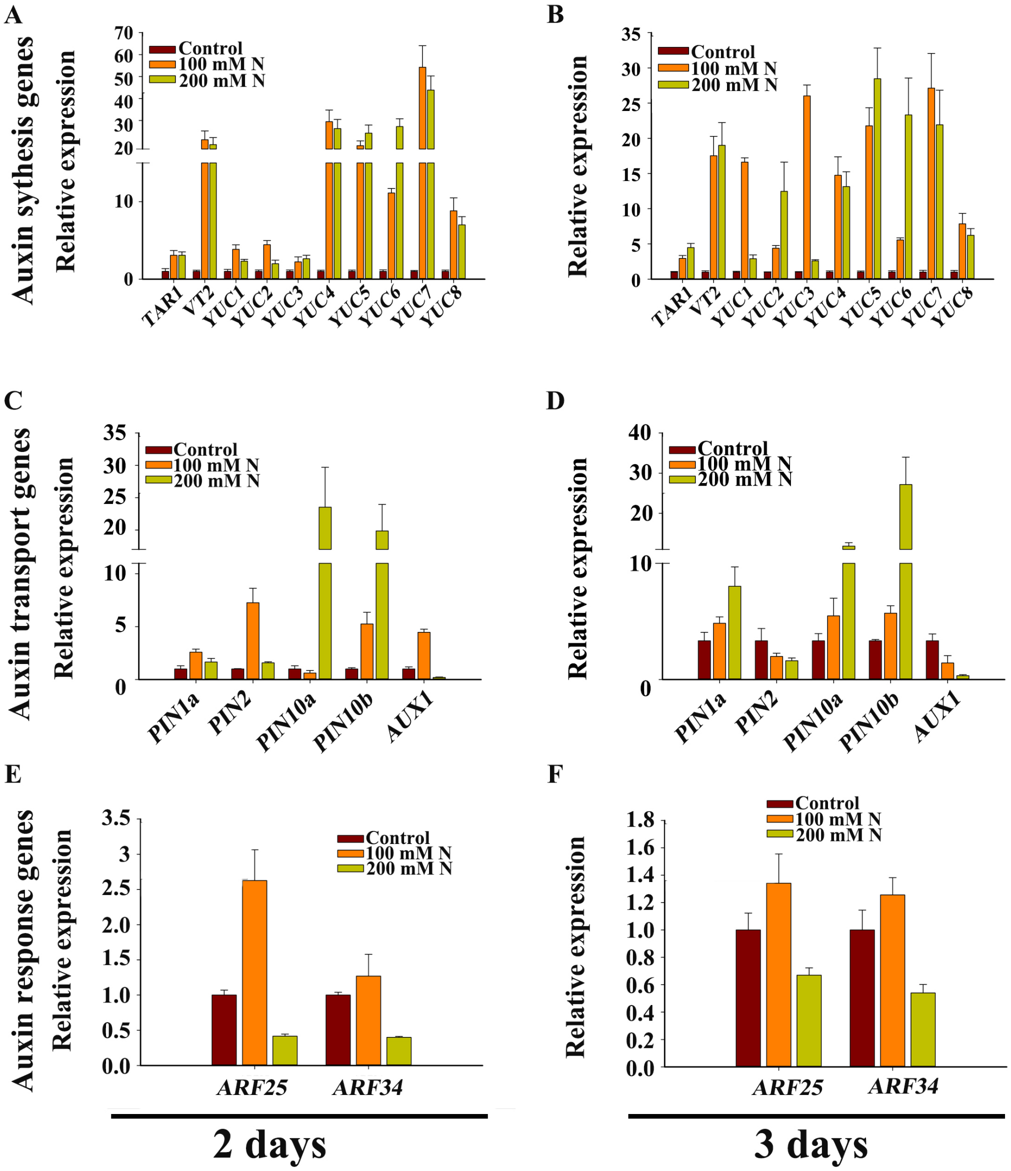

Figure S10 qRT-PCR profiles auixn biosynthesis-, transport-, and degradation genes in response to 100 mM or 200 mM NaCl treatment compares to control.

(A) to (F) Expression levels of auxin synthesis (A and B), auxin transport (C and D), auxin response (E and F) under NaCl treatment for 2 or 3 days. Data represent the means ± SEs of three replicates. N = NaCl.
