# Supplementary material for: Abscisic Acid Regulates Auxin Distribution to Mediate Maize Lateral Root Development Under Salt Stress
Source: Front Plant Sci. 2019 Jun 5;10:716. doi: 10.3389/fpls.2019.00716 (PMC6560076; doi:10.3389/fpls.2019.00716)
Supplement: Supplementary file 4 [file Image_3.pdf]

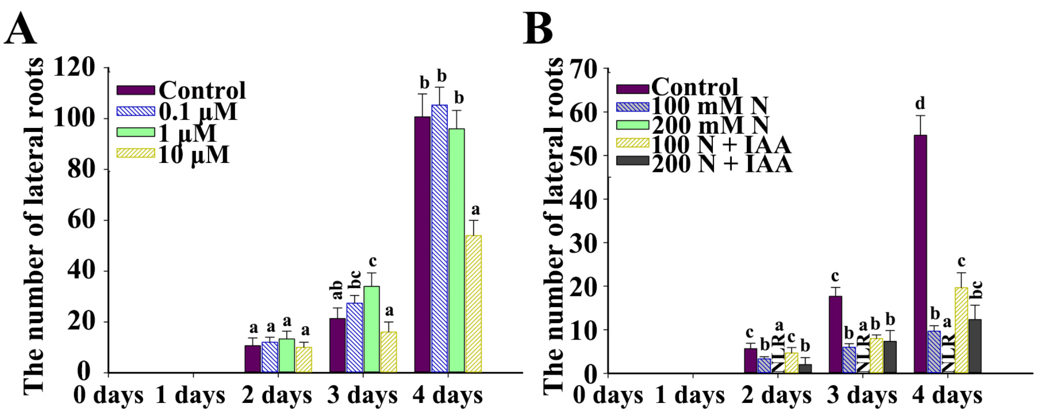

Figure S11 IAA involved in NaCl regulated lateral root development

(A) The number of lateral roots under gradient concentration IAA. IAA treatment concentrations: 0.1 μM, 1 μM, 10 μM.

(B) The number of lateral roots under different treatment. Treatment concentrations: 100 mM NaCl, 200 mM NaCl, 100 mM NaCl + 0.1 μM, 200 mM NaCl + 0.1 μM. N = NaCl

Data represent the means ± SEs of five replicates, with 10 seedlings each in A, and B. Different letters represent significantly differences between treatment and control ($P < 0.05$, based on Student's t test).

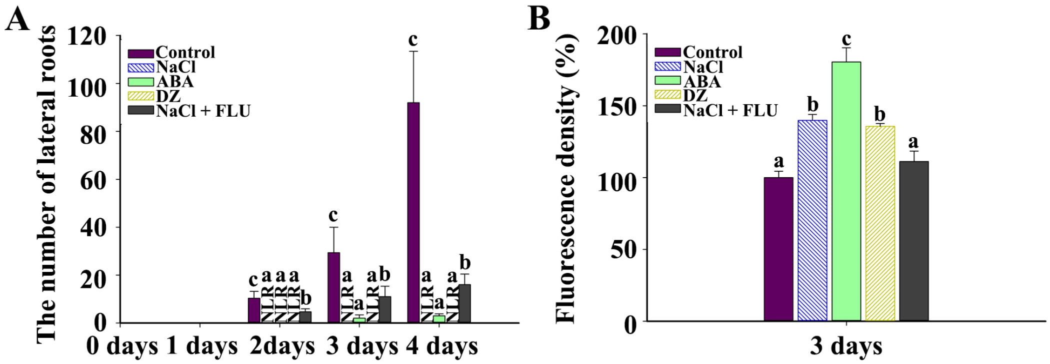

Figure S12 ABA participates in the regulation of lateral root development.

(A) The number of the lateral roots. Wild type (B73), 1 - 4 days after 3-days-old seedlings imbibition in the absence (Hoagland's) or presence of five different treatments. Treatment concentrations: 0 mM NaCl, 200 mM NaCl, 100 μM ABA, 5 μM DZ, 200 mM NaCl + 5 μM FLU.

(B) Fluorescence density of the ABA in transverse root section under different treatments. Treatment concentrations: 0 mM NaCl, 200 mM NaCl, 100 μM ABA, 5 μM DZ, 200 mM NaCl + 5 μM FLU.

Data represent the means ± SEs of five replicates, with 10 seedlings each in A, and B. Different letters represent significantly differences between treatment and control ($P < 0.05$, based on Student's t test).

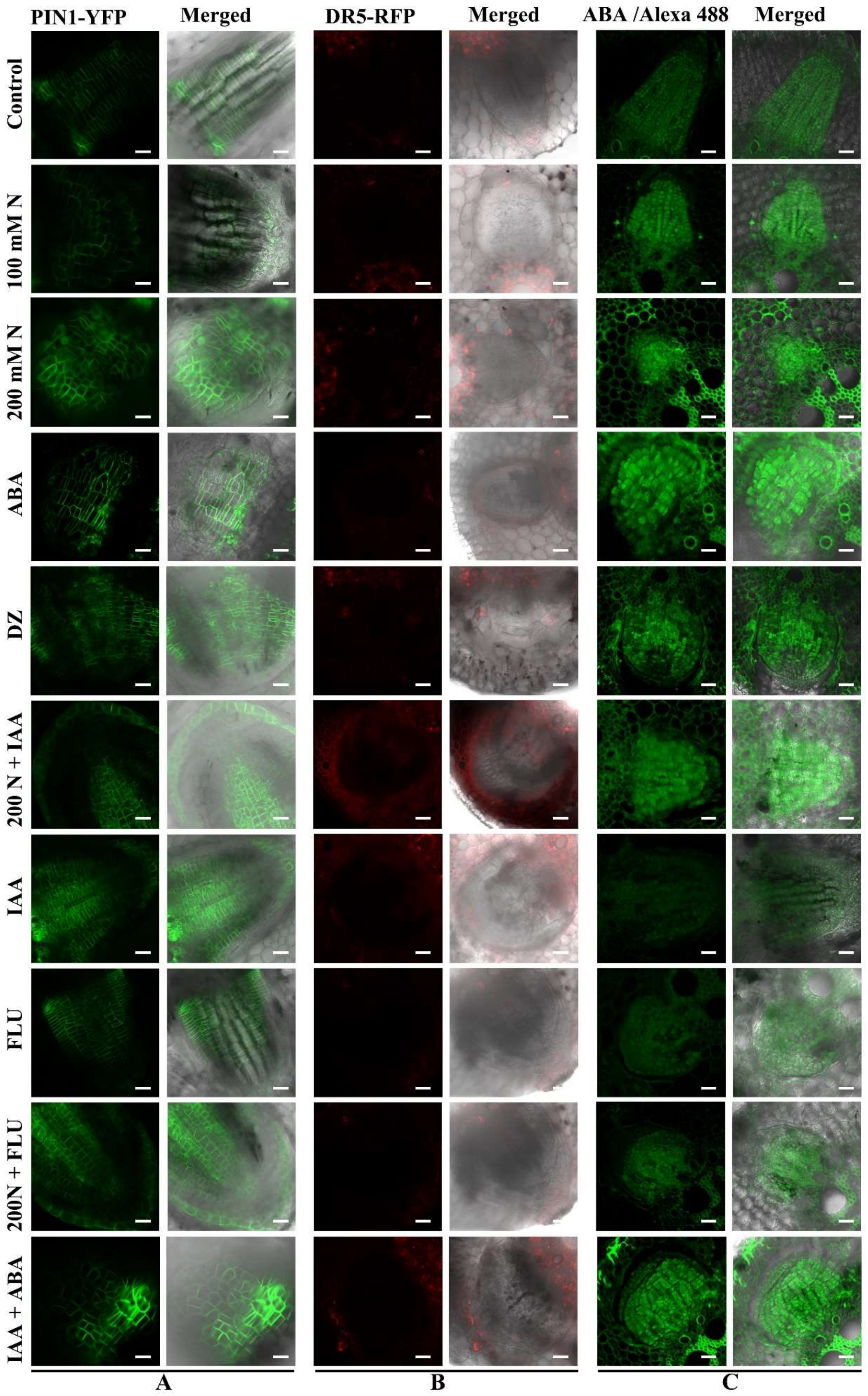

Figure S13 NaCl treatments regulated Auxin distribution via affecting the polar localization of PIN1.

(A) (B) (C) are the transverse section of the LRs. On the left are fluorescent photos, and the right are transmission photos. N = NaCl

(A) Fluorescence of PIN1-YFP under ten different treatment. Bars = 50 μm

(B) Fluorescence of DR5-RFP under ten different treatment. Bars = 50 μm

(C) Immunofluorescence of ABA under ten different treatment. Bars = 50 μm. Treatment concentrations: 0 mM Nacl, 100 mM NaCl, 200 mM NaCl, 100 μM ABA, 5 μM DZ, 200 mM NaCl + 0.1 μM IAA, 0.1 μM IAA, 5 μM FLU, 200 mM Nacl + 5 μM FLU, 0.1 μM IAA + 100 μM ABA.

Photos are selected from five replicates, with 10 seedlings each time.

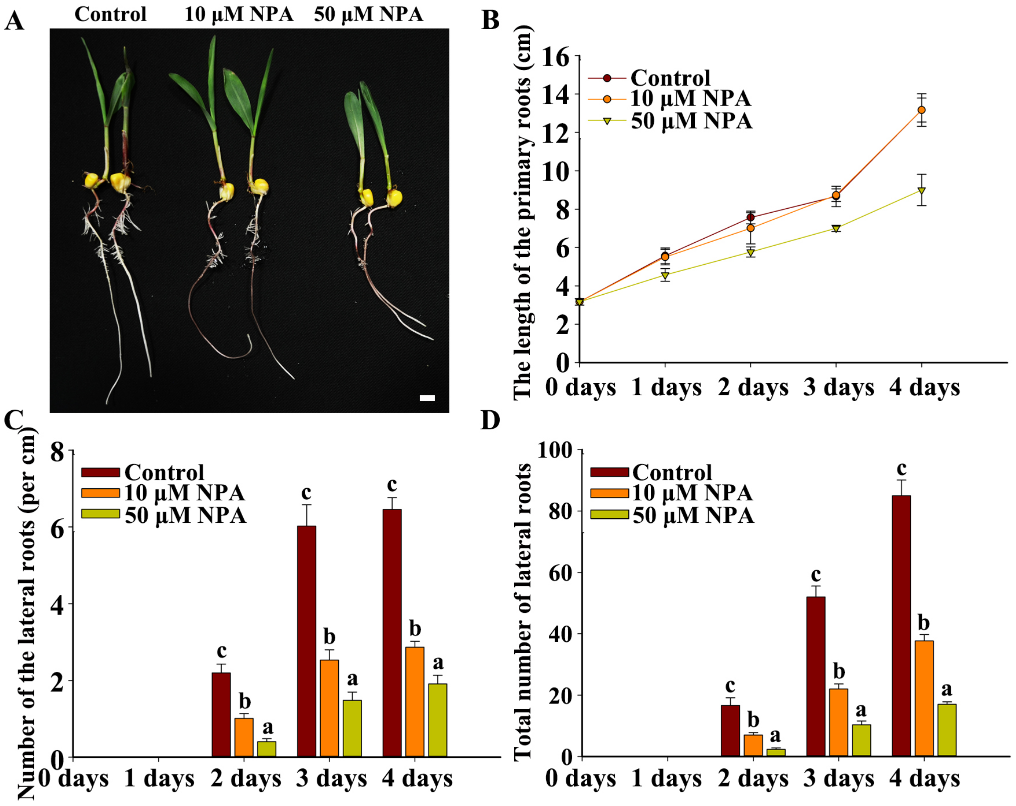

Figure S14 NPA mainly inhibits LRs growth.

(A) Phenotypes of maize under NPA treatment for 4 days. Bar = 1 cm.

(B) The length of PRs from 0 - 4 days under NPA treatment.

(C) LRs density from 0 - 4 days under NPA treatment.

(D) Total number of LRs from 0 - 4 days d under NPA treatment. NPA concentration: 10 µM and 50 µM.

Data represent the means ± SEs of five replicates, with 10 seedlings each in A, and B. Different letters represent significantly differences between treatment and control ($P < 0.05$, based on Student's t test).
